# Continent-wide view of genomic diversity and divergence in the wolves of Asia

**DOI:** 10.1038/s42003-025-09379-9

**Published:** 2025-12-24

**Authors:** Lauren M. Hennelly, Bárbara R. Parreira, Ash Noble, Camilla H. Scharff-Olsen, M. Çisel Kemahlı Aytekin, Çağan H. Şekercioğlu, Pavel Kosintsev, Ladislav Paule, Pavel Hulva, Hans K. Stenøien, Bilal Habib, Hira Fatima, Ghulam Sarwar, Samara P. El-Haddad, Alexandra Youssef, Frank Hailer, Xin Sun, Nuno Filipes Gomes Martins, M. Thomas P. Gilbert, Benjamin N. Sacks, Mikkel-Holger S. Sinding, Shyam Gopalakrishnan

**Affiliations:** 1 Section for Evolutionary Hologenomics, Globe Institute, University of Copenhagen, Copenhagen, Denmark; 2 Center for Conservation Genomics, Smithsonian’s National Zoo and Conservation Biology Institute, Washington, DC USA; 3 Mammalian Ecology and Conservation Unit, Veterinary Genetics Laboratory, Department of Population Health and Reproduction, School of Veterinary Medicine, University of California, Davis, CA USA; 4 Department of BioSciences, Rice University, Houston, TX USA; 5 Organisms and Environment, School of Biosciences, Cardiff University, Cardiff, UK; 6 Department of Molecular Biology and Genetics, Koç University, Istanbul, Türkiye; 7 School of Biological Sciences, University of Utah, Salt Lake City, UT USA; 8 Kuzey Doğa Society, Kars, Türkiye; 9 Ural Federal University, Yekaterinburg, Russia; 10 Institute of Plant and Animal Ecology, Ural Branch of Russian Academy of Science, Yekaterinburg, Russia; 11 Technical University in Zvolen, Zvolen, Slovakia; 12 Department of Zoology, Charles University, Prague, Czechia; 13 Department of Biology and Ecology, University of Ostrava, Ostrava, Czechia; 14 NTNU University Museum, Norwegian University of Science and Technology, Trondheim, Norway; 15 Wildlife Institute of India, Dehradun, India; 16 University of Education – Attock Campus, Attock, Pakistan; 17 University of the Punjab, Lahore, Pakistan; 18 Lebanese Wildlife, Jitta, El Metn Lebanon; 19 Cardiff University-Institute of Zoology Joint Laboratory for Biocomplexity Research (CIBR), Beijing, China; 20 Department of Biology, University of Copenhagen, Copenhagen, Denmark

**Keywords:** Evolutionary genetics, Ecological genetics, Phylogenetics

## Abstract

Grey wolves (*Canis lupus*) in Asia hold most of the species’ genetic diversity and many endangered populations. However, a clear understanding of the evolutionary history of wolves in Asia is lacking, hindering their conservation. We used 98 whole genomes of wolves across Eurasia to better resolve their evolutionary history and conservation status. The strongest barriers to gene flow coincided with boundaries separating the three major wolf lineages - Indian, Tibetan, and Holarctic. Wolves in the central Asian mountain ranges belonged to the Holarctic lineage and share little ancestry with the nearby Tibetan lineage. In contrast, wolves from eastern Asia share population-wide ancestry with the Tibetan lineage, which may reflect an unsampled lineage similar to the Tibetan lineage. Wolves from southwestern Asia share population-wide ancestry with the Indian lineage, likely due to old (>6 kya) admixture events. Long-term declines and recent inbreeding have left Indian and Tibetan wolves with some of the lowest levels of genetic diversity and highest realized genetic loads. In contrast, adjacent populations have some of the highest genetic diversity, due in part to admixture along contact zones. Our study highlights southern regions of Asia as hotspots of wolf diversity and the need to conserve these remaining populations.

## Abstract (web summary)

Wolves in Asia are remarkably diverse, inhabiting some of the world's hottest deserts to the high plains of the Tibetan plateau. However, they remain understudied and poorly understood. Here, we sequenced whole genomes of wolves in Asia to assess their evolutionary history and genetic diversity.

## Introduction

A comprehensive understanding of the evolutionary history of a species is essential to its conservation. Species often consist of distinct populations with genetic variation unevenly distributed across their range. Understanding the contribution of historical and human-induced changes in shaping present-day patterns of genetic variation is one of the key goals in evolutionary biology. This knowledge is especially important for prioritizing populations of concern and informing management of threatened species^1^.

Grey wolves (*Canis lupus*) are one of the most widely distributed mammals, making them an ideal system to study how historical and human-induced factors influence present-day genetic variation. Understanding these influences is particularly important because many wolf populations are endangered or threatened. For instance, some wolf populations in Europe and North America have low genetic diversity due to population declines and inbreeding, like the Mexican and Italian wolves^2,3^. Most studies on the population status and genetic diversity of grey wolves have focused on North American and European portions of their range^3–8^. Fewer studies have focused on wolves in Asia despite the region holding most of the species’ phylogenetic diversity and many endangered populations^9^. The lack of studies in Asia hinders conservation efforts, and a better understanding of how evolutionary distinct populations and genetic diversity vary across Asia can benefit conservation management and decisions.

Recent work has described three divergent wolf lineages—the Indian, Tibetan, and Holarctic lineages, all of which occur in Asia^10–12^ (Fig. S1). These lineages diverged >100,000 years ago, after which the Indian and Tibetan (also called Himalayan) lineages became isolated within separate glacial refugia^10,12^. Wolves from the Holarctic lineage, which span from northern Europe to Siberia, as well as North America, were highly connected over the last 100,000 years, with notable population structure emerging in more recent times^13^. Analyses of ancient wolf genomes suggest wolves across northern Eurasia largely resembled a panmictic population over the last 100,000 years. This extensive genetic connectivity was mainly driven by the western expansion of Siberian ancestry throughout the late Pleistocene until 10,000 years ago^13^. While this recent work has provided a broad picture of wolf evolutionary history in Eurasia, few studies have examined how patterns of ancestry and genomic diversity are distributed across the continent, particularly in more southern regions of Asia, where the three main lineages occur in close proximity.

For example, based on a limited number of genomes, earlier work found wolves from southwestern Asia (defined as the region spanning Iran to the Arabian Peninsula and Türkiye) reflected Holarctic ancestry with minor ancestry from more divergent taxa, such as the Indian wolf and another canid species, the African wolf (*Canis lupaster*)^11,14,15^. Genomic analyses have confirmed a hybrid containing African wolf, grey wolf, and domestic dog ancestry from the Sinai Peninsula of Egypt^14^. Additionally, hybrids between the African wolves and dogs have been genetically confirmed from Senegal and Ethiopia, suggesting the two different species can interbreed^16^. However, a fuller understanding of wolves from southwestern Asia has been hampered by a lack of geographically representative samples from across this region. For wolves from eastern China and extinct wolves from Japan, previous work found they contain minor ancestry attributable to ancient Pleistocene wolves from ~35–50 thousand years ago (kya)^17–19^. In addition, both populations retain ancestry that is more divergent than ancient wolves from northern Eurasia that date back to 100 kya^13^. Together, these findings suggest various wolf populations in southern regions of Asia may retain ancestry distinct from northern Eurasian wolves.

We sought to address four gaps in our knowledge about wolves in Asia through building a genomic dataset that included 20 newly sequenced wolves sampled from key regions and 81 previously published wolf genomes from Eurasia and North America. Our objectives were to (1) characterize continental-level population structure and gene flow patterns of modern-day wolf populations across Eurasia, (2) clarify the evolutionary history of wolf populations in the central Asian mountain ranges and eastern regions of Asia (3) clarify the evolutionary history of wolves in southwestern Asia, and (4) characterize the genetic diversity, inbreeding levels, and genetic load of wolves throughout Eurasia to understand long-term and recent impacts on genetic diversity.

## Results

For this study, we resequenced new whole genomes of 20 wolves from various regions in Eurasia. These regions included southwestern Asia (Lebanon, *n* = 1; Israel, *n* = 1, Türkiye=1), Indus plains of Pakistan (*n* = 2), central Asian mountain ranges (Sulieman, *n* = 2; Karakoram, *n* = 2; Hindu Kush, *n* = 3), the Eurasian steppe (Kazakhstan, *n* = 3; Russia, *n* = 3), and in Europe (Slovakia, *n* = 1; Ukraine, *n* = 1). We also conducted additional (i.e., deeper) sequencing of a previously sequenced Indian wolf from Central India. We compiled a dataset with these newly sequenced genomes and 95 previously sequenced wolves from North America (*n* = 3) and Eurasia (*n* = 78), as well as dogs (*n* = 4), and 10 individuals from six other canid species: dhole, *Cuon alpinus* (*n* = 1); Ethiopian wolf, *Canis simensis* (*n* = 1); golden jackal, *Canis aureus* (*n* = 1); African wolf, *Canis lupaster* (*n* = 4); coyote, *Canis latrans* (*n* = 2), Andean fox; *Lycalopex culpaeus* (*n* = 1). This resulted in a total of 115 samples included in the study, which we aligned to the domestic dog genome assembly (canFam3.1) (Supplementary Data 1).

### Population structure and gene flow patterns of wolves in Eurasia

**Fig. 1:**
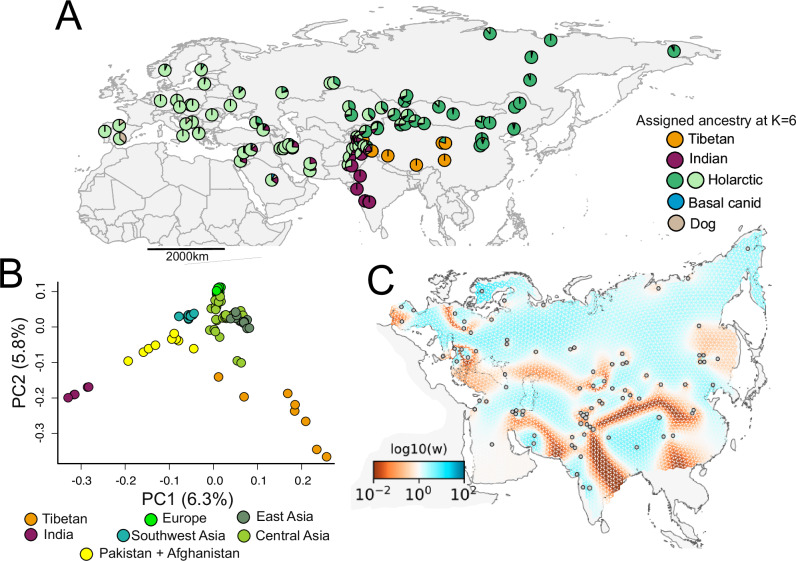
Population genetic structure and genetic connectivity of wolves across Eurasia. **A** Sample location and individual admixture proportions at *K* = 6 for 98 wolves across Eurasia. The admixture proportions were estimated using 98 Eurasian grey wolves, 3 North American wolves, 4 dogs, and 10 individuals from six other canid species. **B** Principal component analysis using genotype likelihoods for 98 wolves across Eurasia. Colors indicate the geographic region to which each individual wolf belongs. **C** The estimated effective migration surface (EEMS) for 97 wolves across Eurasia. Colors of the EEMS correspond to lower-than-average (red) and higher-than-average (blue) effective migration as expressed as log_10_(*w*). A lambda of 100 was used after performing cross-validation analyses.

Principal component analysis (PCA) and inferred individual admixture proportions of 98 Eurasian wolves supported the presence of three major clusters corresponding to the three major extant wolf lineages in Eurasia: the Indian, Tibetan, and Holarctic wolves (Fig. 1a, b, Supplementary Figs. 2 and 3). Some wolves fell in-between these main clusters, forming clines between Tibetan and Holarctic as well as between Indian and Holarctic clusters. In both cases, these intermediate individuals came from areas near the corresponding contact zones between lineages. Intermediate individuals on the Tibetan-Holarctic cline included two wolves from Tajikistan (~24% Tibetan ancestry at *K* = 6) and two wolves from Ladakh, which is a region on the southwestern edge of the Tibetan plateau (Supplementary Fig. 2). Intermediate individuals on the Indian-Holarctic cline included only wolves from Pakistan (Supplementary Fig. 4). In contrast, all 13 wolves from southwestern Asia clustered tightly together nearby to other Holarctic wolves and exhibited ~15–20% Indian wolf ancestry at *K* = 6. A few wolves, most sampled near Africa, showed ancestry that did not cluster with grey wolves and dogs, possibly reflecting admixture from the African wolf.

To better characterize geographic patterns of gene flow, we inferred the estimated effective migration surface (EEMS), which indicates regions of low or high gene flow relative to an isolation-by-distance model^20^. Mirroring the PCA and admixture results, the greatest EEMS barriers to gene flow in Eurasia corresponded to divisions among the three major lineages (Fig. 1c). The strongest barrier was along the Himalayan mountain range that separates the Indian and Tibetan lineages. A second strong barrier surrounds the Tibetan plateau, in agreement with our PCA and admixture results showing reduced gene flow between the Tibetan lineage and wolves in central and eastern Asia. We detected another barrier to gene flow positioned west of Pakistan; however, the exact position of the barrier is less clear due to a lack of sampling on the Eurasian steppe, such as in Turkmenistan and Uzbekistan. Otherwise, the population structure of Holarctic wolves across northern Eurasia can be modeled as isolation-by-distance (Fig. 1b, c)

### Evolutionary history of wolves in the central Asian mountains and eastern Asia

**Fig. 2:**
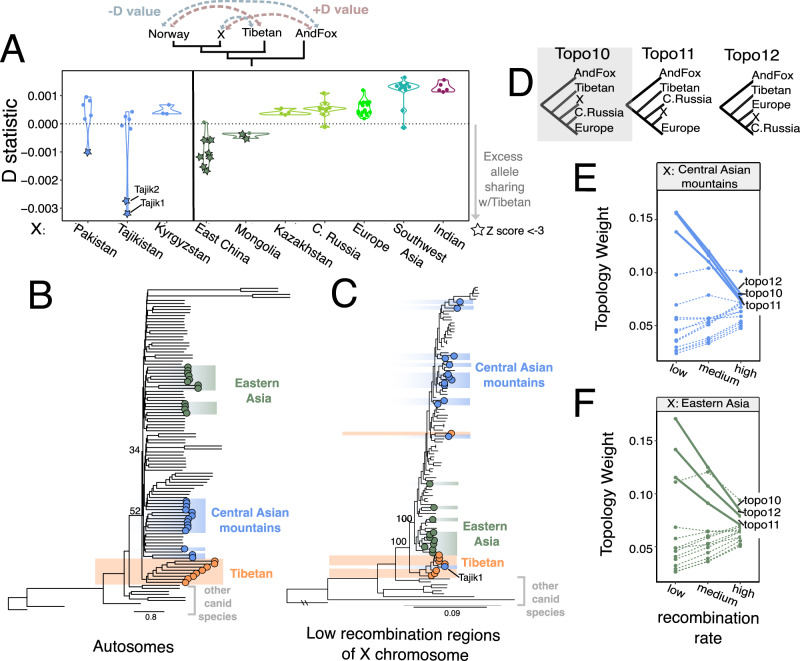
Evolutionary history of wolves from central Asia and eastern Asia. **A** *D* statistic results in estimating derived allele sharing between wolf individuals in Eurasia and the Tibetan lineage. The only populations that show significant allele sharing with Tibetan wolves are some wolves from Pakistan, Tajikistan, Mongolia, and eastern China. **B** Autosomal phylogeny using 97 wolves from Eurasia, 3 North American gray wolves, and 9 individuals from five other canid species. The coalescent-based tree was estimated using 1000 regions of 20 kb length that were randomly chosen across the dog reference genome. The normalized quartet score of selected nodes is shown. Colored circles and shaded color within the phylogeny indicate the individuals assigned to the Tibetan lineage (orange), central Asian mountains (high altitude Pakistan, Tajikistan, Kyrgyzstan; blue), and eastern Asia (eastern China, Mongolia, eastern Russia; green). **C** A maximum-likelihood tree inferred with only low recombination (<0.2 cM/Mb) regions of the X chromosome using the same sample set as in (**B**). Local posterior probability is labeled at selected nodes. **D** The three topologies with the highest average topology weightings across the X chromosome when considering wolves from the central Asian mountains or eastern Asia as the focal population X. The topology shaded in gray depicts a scenario where the focal wolf population is ancestral to wolves from Europe and Central Russia. Prevalence of 15 possible topologies in high, medium, and low recombination regions on the X chromosome where the focal population is from **E** central Asian mountains and **F** eastern Asia. For both central Asian and eastern Asian wolves, three topologies (solid lines) show a negative relationship between recombination rate and topology weighting, in contrast to the 12 other topologies (dotted lines).

To explore the evolutionary history of wolves in the central Asian mountain ranges and eastern Asia, we first used *D* statistics to test for excess derived allele sharing with various wolf populations in Asia^21^. We observed wolves from both regions had a significant excess (*Z* score < −3) of derived alleles shared with the Tibetan lineage, and not with any other taxa (Indian wolf, African wolf, golden jackal) (Supplementary Figs. 5–8). However, for wolves in the central Asian mountains, the extent of allele sharing with the Tibetan lineage varied widely among individuals (Fig. 2a). Only three out of 16 wolves showed significant allele sharing with the Tibetan lineage, in which all wolves from Kyrgyzstan and most wolves from Pakistan and Tajikistan had no detectable allele sharing with the Tibetan lineage (Fig. 2a; Supplementary Fig. 5). This high inter-individual variance in Tibetan derived alleles suggests gene flow with Tibetan wolves is limited, sporadic, and/or possibly recent. In contrast to wolves from central Asian mountains, we observe a consistent and significant excess of allele sharing with the Tibetan lineage for almost all wolves found in eastern China and Mongolia (Fig. 2a, Supplementary Figs. 5, 9).

It can be difficult to identify the actual source of ancestry using *D* statistics, because any ancestry that is as divergent as the Tibetan wolf will result in a negative *D* statistic. To better understand the source of ancestry within wolves of eastern Asia, we inferred a set of phylogenetic trees from different regions of the genome. We first inferred a multispecies coalescent tree in ASTRAL using 1000 regions with a length of 20 kb across the autosomes of 107 individuals^22^. We found Tibetan wolves formed the earliest diverging lineage within grey wolves (Fig. 2b). The rest of the wolves in Eurasia and North America clustered together, including Indian wolves, which are clustered with wolves from Pakistan and sister to those from southwestern Asia (for a detailed tree with sample names, see Supplementary Fig. 10).

Because genome-wide phylogenies can be influenced by processes such as gene flow and incomplete lineage sorting (ILS), we leveraged how genome architecture relates to phylogenetic signal^23^. Briefly, the X chromosome and low recombination regions are more likely to retain the historical relationship due to differences in inheritance, effective population size (*N*_e_), and selection^24–26^. The X chromosome has a smaller *N*_e_ since males only carry one copy, resulting in faster lineage sorting and, in the case of male-mediated gene flow, may better retain the historical relationship^27–30^. Low recombination regions experience stronger selection against introgressed ancestry due to higher linkage and a lower *N*_e_^24,31^. Therefore, we inferred phylogenetic trees using the X chromosome and low recombination regions to take advantage of their association with the historical phylogenetic relationship, as demonstrated in multiple taxa, including wolves^11,32–36^.

Our phylogenetic trees based on low-recombination regions (<0.2 cM/Mb) of the X chromosome and autosomes demonstrated that wolves from the Tibetan plateau form the earliest diverging branch within grey wolves. In contrast, Indian wolves clustered with wolves from Pakistan and southwestern Asia, a result that could be due to admixture between these populations (Fig. 2c, Supplementary Fig. 11). Interestingly, one of the two wolves from Tajikistan with the highest allele sharing with Tibetan wolves clustered within the Tibetan lineage, and all other wolves from Tajikistan and Kyrgyzstan clustered within the Holarctic lineage (Fig. 2c, for a detailed tree see Supplementary Fig. 12). Wolves from eastern Asia formed a well-supported clade that branched basally to the rest of the Holarctic lineage for the X chromosome phylogeny and phylogenies using low-recombination regions (Fig. 2c, Supplementary Figs. 11, 13). In addition, topology weighting analyses revealed topologies where eastern Asian wolves form the earliest diverging branch within the Holarctic lineage are more prevalent in low recombination regions of the X chromosome (Fig. 2d–f, Supplementary Fig. 14). Thus, while wolves in Tajikistan, Pakistan, and eastern Asia have similar *D*-values indicating significant derived allele sharing with Tibetan wolves, they have contrasting topological patterns. This is consistent with wolves from Tajikistan and Pakistan having experienced recent and/or periodic gene flow from Tibetan wolves near the secondary contact zone; however, this Tibetan ancestry has not spread widely through the population. In contrast, wolves in eastern China and Mongolia may have retained population-wide ancestry that is more divergent than wolves in northern Eurasia and similar to modern-day Tibetan wolves.

### Complex history of admixture within the wolves of southwestern Asia

**Fig. 3:**
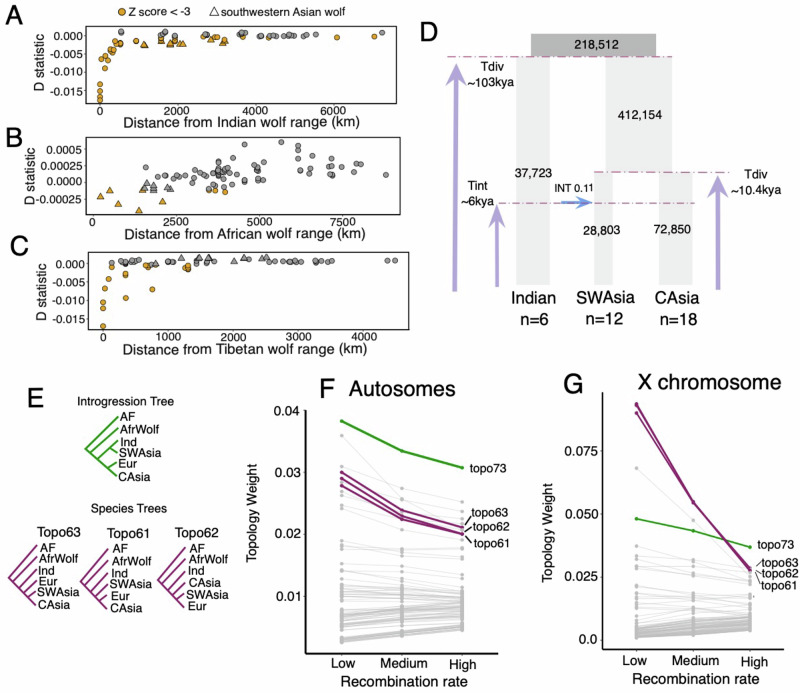
Complex history of admixture within the wolves of southwest Asia. *D* statistic values plotted against geographic distance away from the **A** Indian wolf, **B** African wolf, and **C** Tibetan wolf distributional ranges, as defined in Fig. S1. A negative *D* statistic indicates increased derived allele sharing with the **A** Indian wolf, **B** African wolf, and **C** Tibetan wolf (**A**) as X based on the topology (((Norway wolf, Y), X), Andean fox). **D** The model with the highest likelihood inferred using fastsimcoal2 corresponds to an introgression event between the Indian lineage and wolves of southwestern Asia. Time is given in years and was obtained by assuming a generation time of 4.4 years. **E** The topologies representing the introgression scenario (green), historical relationship (purple), and other processes such as ILS (gray) that were used to infer topology weights when using an outgroup (Andean fox, AF), the African wolf (AfrWolf), and gray wolf populations from India (Ind), southwestern Asia (SWAsia), central Asia (CAsia), and Europe (Eur). Average topology weights for each of 105 possible topologies found in low (<0.2 cM/Mb), medium (0.2–2 cM/Mb), and high (>2 cM/Mb) recombination rate regions for all 105 possible topologies across the **F** autosomes and **G** X chromosome.

Our estimated admixture proportions and *D* statistics suggest wolves in southwestern Asia shared ancestry with the Indian lineage and another canid species, the African wolf (Supplementary Figs. 2, 6, and 7). We detected nine wolves that showed significant allele sharing with the African wolf and these wolves were largely confined to areas most geographically close to the distributional extent of the African wolf range in the Sinai Peninsula of Egypt (Fig. 3b). We also detected one wolf from Syria having significant derived allele sharing with the golden jackal, as well as African wolf and the Indian wolf (Supplementary Fig. 8). Overall, our *D* statistic results suggest the divergent ancestry present in these nine individuals is most similar to the African wolf, as found in earlier studies^14^.

Interestingly, we found ancestry shared with the Indian lineage was nearly equal in all wolves in southwestern Asia (Fig. 3a, Supplementary Figs. 6, 9, 15). There appeared to be no decline in the admixture proportions of assigned Indian ancestry at *K* = 6 from the contact zone in Pakistan until reaching the Caucasus mountain range and from Türkiye into Europe (Supplementary Fig. 2). There was also a lesser, yet significant (*Z* score < −3), signal of shared ancestry with the Indian lineage on parts of the Eurasian steppe in Central Asia, such as in Kazakhstan (Supplementary Figs. 6,9). This geographically widespread signal contrasts with what we observed with Tibetan and African wolf ancestry, which declines quickly as distance increases from their respective distributions (Fig. 3b, c).

The balanced Indian wolf ancestry across southwestern Asia could be consistent with a historical admixture event between Indian and Holarctic lineages. To investigate the origin of Indian ancestry, we used fastsimcoal2^37^ to test three demographic scenarios: (A) no introgression between Indian wolves and southwestern Asian wolves, (B) southwestern Asian wolves experienced an introgression event with Indian wolves, and (C) southwestern Asian wolves formed as a hybrid lineage between Indian and central Asian wolves (Supplementary Fig. 16). We obtained similar likelihood scores for the three models, suggesting no one model was strongly favored over the others (Supplementary Table 1). However, topologies that included bifurcation (models A and B) had the highest likelihoods. The model that included introgression from Indian wolves to southwestern Asian wolves (B) was preferred, with a relative likelihood of 62% (Supplementary Table 1). Estimates suggest a relatively high introgression proportion (0.11) from India to southwestern Asia, consistent with our *D* statistic results (Fig. 2d). We estimated that introgression occurred around 6000 years ago, relatively close to the divergence time between southwestern and central Asian populations about 10,300 years ago. Therefore, if introgression did occur, it likely happened near the time of the split between southwestern and central Asian populations, explaining the similar likelihood between the divergence (A) and introgression (B) models. Importantly, both models estimate similar dates for the split between southwestern and central Asian populations, and all models date the divergence of Indian wolves from Eurasian wolves to around 100,000 years before present, providing robustness to time estimates. Additionally, both introgression and divergence models produced similar estimates for population sizes, suggesting a small effective population size (*N*_E_) for wolf populations in India and southwestern Asia, whereas central Asian populations and the ancestral Holarctic population in Asia had a larger *N*_E_ (Fig. 16). Our results indicate that the Indian ancestry within southwestern Asian wolves reflects older admixture (>6 kya) and/or shared demographic history rather than due to recent gene flow in the last thousands of years.

Finally, we inferred a set of phylogenetic trees to investigate the evolutionary history of southwestern Asian wolves in more detail. In both the autosomal tree and the X chromosome tree, we observe the Indian lineage and wolves from Pakistan and southwestern Asian wolves clustering as sister clades—nested all within the Holarctic clade (Supplementary Figs. 10 and 13). However, when excluding wolves from Pakistan and southwestern Asia, Indian wolves form the earliest diverging branch of the grey wolf phylogeny (Supplementary Fig. 16). This pattern is consistent with minor Indian wolf ancestry within southwestern Asian wolf genomes, causing phylogenetic affinity, resulting in an apparent sister relationship between the two taxa when forced into a bifurcating tree^12^ (Supplementary Fig. 17).

To better investigate how phylogenetic signal is distributed across the genome, we quantified the frequency of 105 possible topologies using 100-SNP windows consisting of wolves from India, southwestern Asia, Europe, and central Asia, the African wolf, and the Andean fox as an outgroup (Fig. 3e). The most frequent topology across the autosomes reflected introgression in which southwestern Asian wolves were sister to Indian wolves, including in low recombination regions (<0.2 cM/Mb) (Fig. 3g). The historical relationship, in which the Indian lineage is the earliest diverged, were among the highest weighted topologies and when considering all three topologies together, are the most prevalent phylogenetic signal in the autosomes. For the X chromosome, all topologies representing the historical relationship were most prevalent, including within low recombination regions (Fig. 3g). Unlike eastern Asian wolves, which consistently formed an earlier diverged clade within Holarctic wolves, our results indicate southwestern Asian wolves show two primary topological patterns: sister to the Indian lineage and clustered within the Holarctic clade. This may suggest southwestern Asian wolves share much of their evolutionary history with northern Eurasian wolves and derive part of their ancestry from gene flow with an Indian wolf population similar to those found today.

### Historical and present-day impacts on genetic diversity

**Fig. 4:**
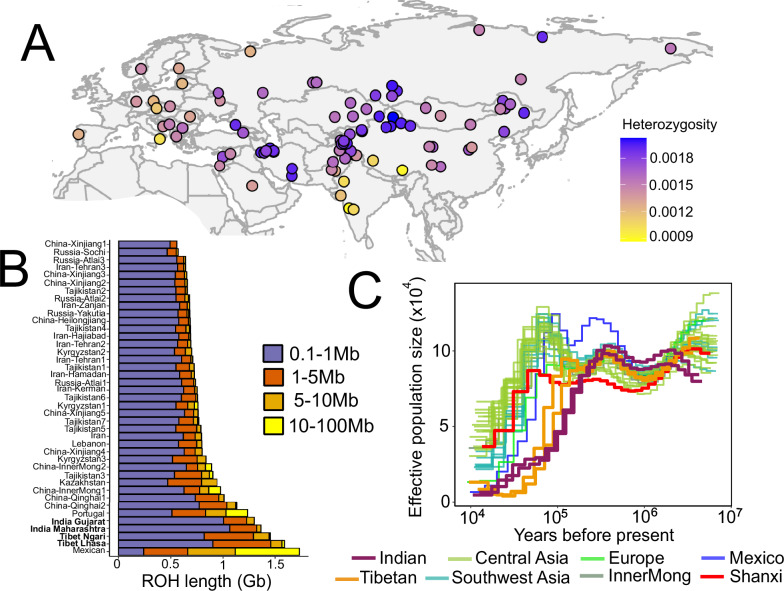
Recent and long-term demographic history of wolves in Eurasia. **A** Estimates of genome-wide heterozygosity for 96 wolves from Eurasia. **B** Runs of homozygosity (ROH) for 41 wolves showing the sum of the length of ROH in four size ranges: 0.1–1 Mb (Short ROH), 1–5 Mb (Short-medium ROH), 5–10 Mb (medium-long ROH), and 10–100 Mb (long ROH). Individuals are arranged from top to bottom by the sum length of ROH, with wolves representing the Indian and Tibetan lineages bolded. **C** Pairwise sequentially Markovian coalescent (PSMC) analysis of the demographic histories for 32 wolves from Eurasia and North America.

Our whole-genome dataset allowed us to assess patterns of genome-wide diversity of wolves across Eurasia and North America. We estimated autosomal heterozygosity using genotype-likelihoods for 97 wolves from Eurasia (*n* = 94) and North America (*n* = 3), and found wolves in Asia possess some of the lowest and highest levels of genetic diversity in our dataset (Fig. 4a). In addition to the well-known depleted heterozygosity in Mexican and Iberian wolves, we found exceptionally low heterozygosity in wolves from India and the Tibetan plateau. As observed in our pairwise sequentially Markovian coalescent (PSMC) analysis, this low heterozygosity is in part due to long-term population declines and/or a lack of genetic connectivity over the past >100 kya^38,39^. We also observe that the wolf from Shanxi, China, had a lower population size throughout the Pleistocene compared to other Holarctic wolves (Fig. 4c). This may suggest the Shanxi wolf and other wolves in southern China were less connected to northern wolf populations, however, a greater number of higher coverage genomes are needed to rule out the effects of low coverage and limited sampling. Otherwise, there is a shift to elevated heterozygosity coinciding with secondary contact zones between the Holarctic lineage and the Indian and Tibetan lineages, respectively. For example, admixed wolves in lowland Pakistan had higher genome-wide heterozygosity than wolves of Central India, even though both populations are similarly endangered and declining. We found that the highest heterozygosity among wolves in Asia is geographically clustered in Iran and Xinjiang of China (Fig. 4a). These findings are concordant with our estimated inbreeding coefficients using genotype likelihoods^40^ (Supplementary Fig. 18).

To further disentangle the historical and present-day processes shaping genetic diversity in wolves, we inferred runs of homozygosity (ROH) for genomes with coverage above 15× using BCFtools/RoH^41^. The lengths of ROHs allowed additional insight into the timing of inbreeding. Combined with knowledge of the generation time and assuming a constant recombination rate across the genome, we estimated the timing of inbreeding, where, for example, a ROH length of 5 Mb suggests inbreeding took place around 7 generations ago^42^. We find that Indian and Tibetan wolves show the most extensive inbreeding around 800–102 years ago compared to all other wolves in our dataset (Supplementary Fig. 19). Along with the highest number of short ROHs (0.1–1 Mb), Indian and Tibetan wolves show evidence of larger ROH as well (5–10 Mb), suggesting their low heterozygosity is a combination of long-term isolation over the last >100 kya and more recent declines in the last hundreds of years (Fig. 4b, Supplementary Fig. 19). We find the well documented and dramatic population declines of Mexican and Iberian wolves occurred more recently over the course of the last century, in line with previous work^7,43^ (Supplementary Fig. 19). Overall, our results suggest much of the variation in genetic diversity in Asia can be explained by historical processes, including long-term isolation, shifts in connectivity coinciding with glacial refugia and secondary contact zones.

### Genetic load in wolf populations across Asia

**Fig. 5:**
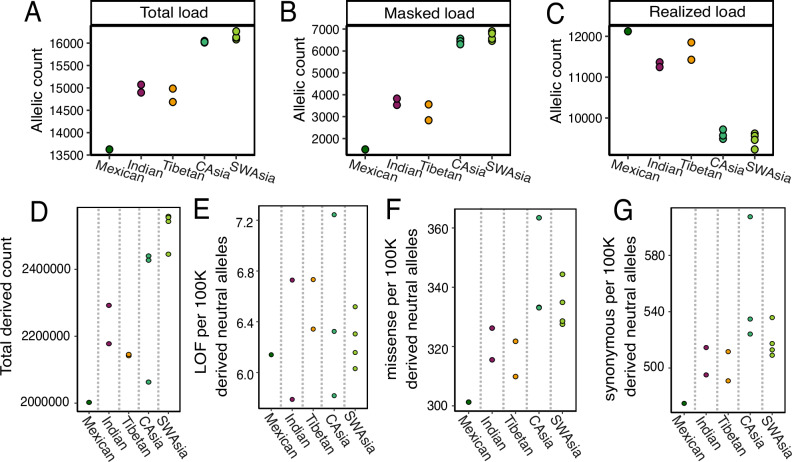
Genetic load in selected wolf individuals in Asia and North America. **A** Total genetic load, **B** masked load, and **C** realized load for 13 wolf individuals within five populations. Wolves found in historically smaller populations (Mexican, Indian, and Tibetan) have a lower total load, lower masked load, and higher realized load. The total derived allele count of each wolf within the five populations, where generally Mexican, Indian, and Tibetan wolves show overall less derived alleles. The derived allele count of **D** includes all derived alleles, **E** Loss of function (LOF) mutations classified as high impact, **F** missense mutation classified as moderate impact, and **G** synonymous mutations classified as low impact per 100,000 derived alleles found in non-coding regions, assumed to be neutral. When taking into account differences in the overall number of derived counts, Mexican, Indian, and Tibetan wolves show a reduced number of missense and synonymous mutations, but not for loss-of-function mutations, compared to central Asian and southwest Asian wolves.

Because Indian and Tibetan wolves show low genetic diversity, we explored how another component of genetic diversity—functional genetic diversity— compares to other wolves across Asia. Specifically, we quantified patterns of putative deleterious variation, which is expected to have a negative impact on fitness. Using SnpEff to estimate functional impact of mutations, we quantified the total genetic load (total number of derived alleles classified as high or moderate impact), realized load (homozygous state of derived alleles in high or moderate impact), and masked load (heterozygous state of derived alleles in high or moderate impact) for five selected wolf populations with assuming the derived alleles was the deleterious one^44,45^. Wolves from historically larger population sizes—central and southwestern Asia—had higher masked and total genetic load than wolves from historically smaller populations (Indian, Tibetan, Mexican wolves)^46^ (Fig. 5a, b, Supplementary Figs. 20, 21). This is consistent with theoretical predictions that larger populations with higher genetic diversity should also possess more deleterious alleles in a heterozygous state^47^. In contrast, we found that Indian, Tibetan, and Mexican wolves have a higher realized load and a lower total genetic load (Fig. 5c) than the central Asian and southwestern Asian populations. A higher realized load in these small populations is expected because genetic drift and inbreeding will result in an increased proportion of homozygous genotypes, including deleterious ones.

Next, we conducted additional analyses to explore whether the lower total genetic load in the Indian and Tibetan wolves can be attributed to a loss of derived variants due to genetic drift in a historically small population and/or purging, whereby purifying selection removes recessive deleterious alleles in a homozygous state^48^. To look for evidence of genetic drift versus purging, we asked whether deleterious alleles are lost at a higher frequency than the background neutral alleles (mutations in non-coding regions) for each individual wolf. In other words, does the fact that derived alleles are categorized as deleterious result in more frequent loss compared to neutral derived alleles within an individual, as expected for purging? Or are deleterious derived alleles lost at the same frequency as neutral derived alleles, suggesting the loss in deleterious derived alleles found in our total genetic load results could be due to genetic drift? We assessed the prevalence of sites associated with four impact categories: high impact (loss of function, LOF), moderate impact (missense), low impact (synonymous), and modifier (non-coding; here assumed to be selectively neutral). To account for differences in total derived alleles found in wolf individuals, we divided the total number of LOF, missense, and synonymous derived mutations by the total number of neutral derived alleles assumed to be under neutral processes (mutations in non-coding regions) for each individual.

We found some evidence that a lower total genetic load in Indian, Mexican, and Tibetan wolves may be due to genetic drift rather than purging via purifying selection; however, we suggest caution in interpreting our genetic load results. We do not observe Indian, Tibetan, and Mexican wolves having proportionally fewer high-impact mutations than wolves in central and southwestern Asia (Supplementary Fig. 5e). This observation suggests that genetic drift, rather than purging, may have removed putative strongly deleterious alleles at frequencies comparable to those of neutral derived alleles. Another reason for these results could be that LOF mutations were incorrectly annotated in the short-read assembled dog reference, as suggested by Smeds et al. (2023)^49^. In addition, among the LOF mutations that may be correct, there are so few LOF sites across the genome (*n* = 145 on average in our dataset), and being such a small number, their frequency may be more strongly influenced by genetic drift rather than purifying selection. For moderate and low impact mutations, we found some support that purging via purifying selection may have differentially removed moderate and low impact mutations. Specifically, we found Indian, Tibetan, and Mexican wolves have proportionally fewer missense and synonymous mutations per 100,000 derived neutral mutations compared to wolves in central and southwest Asia (Supplementary Fig. 5f, g). However, we emphasize that it is difficult to differentiate evidence of genetic drift and purging, and further work is needed to verify the functional impacts of our missense and synonymous mutations. Overall, we suggest caution when interpreting our genetic load results. Further work is needed to assess the accuracy of annotations from a short-read assembled genome, the functional impacts of mutations, and to increase the number of individuals included in each population.

## Discussion

We present a comprehensive whole-genome analysis of population structure, patterns of divergence, and genomic diversity of wolves in Asia. We find that the strongest barriers to gene flow in Eurasia coincide with contact zones among the Indian, Tibetan, and Holarctic lineage distributions. We show that wolf populations found in southern regions of Asia retain ancestries not found in Northern Eurasia, including components with some of these ancestries being similar to the African wolf, as well as Indian and Tibetan lineages. In contrast to the recurrent gene flow across northern Eurasia during the late Pleistocene, our work suggests this high connectivity did not completely replace existing wolf ancestry in southern regions of Asia^13^. Lastly, we find that broad-scale patterns of genetic diversity of wolves in Asia are largely explained by historical processes, such as long-term isolation within glacial refugia and contact zones.

Eurasia is the biogeographic cradle for grey wolf evolution, so it is not too surprising that southern regions of Asia have greater evolutionary diversity^13,50,51^. Previous work has also shown that the southern regions of Asia contain ancestry that is more divergent than modern and late Pleistocene wolves from northern Eurasia^13,18,19,52^. For instance, present-day wolves from southwestern Asia, eastern parts of China, and extinct wolves from Japan contain ancestry that is more divergent than a 100 kya ancient wolf genome from Siberia^13^. For eastern Asian wolves, we detected significant-derived allele sharing with the Tibetan lineage as P3, but not with the Indian lineage, African wolf, or golden jackal as P3 (Supplementary Figs. 5–8). While this suggests the origin of this ancestry within eastern Asian wolves is similar to the modern-day Tibetan lineage, the degree to which this ancestry is similar remains unclear. Our phylogenetic trees provide some clarity. Neither the X chromosome phylogenetic tree nor most frequent topologies using twisst showed eastern Asian wolves clustering within or sister to the Tibetan lineage (Fig. 2f, Supplementary Fig. 14), as expected if gene flow between eastern Asian wolves and Tibetan wolves occurred recently^53^. Instead, our results showed eastern Asian wolves forming an independent lineage that diverged before the rest of the Holarctic wolves, yet after the Tibetan lineage. Their early diverging position is most evident with the X chromosome and low recombination phylogenies, a pattern that may reflect historical population structure during the late Pleistocene^30^. Thus, we hypothesize that wolves in eastern parts of Asia may have partial ancestry closely related but distinct to modern-day Tibetan wolves and were not fully replaced by extensive gene flow with northern Holarctic wolves during the late Pleistocene.

Consistent with this, previous work found historical genomes of wolves in southern China and Tibetan wolves occupy opposite ends of the PC2 axis, suggesting wolves from southern China may possess distinct ancestry from the Tibetan lineage^54^. In addition, a ~100,000 year old mitochondrial genome of a wolf from Eastern Asia represented a previously unidentified maternal lineage that diverged before other Holarctic wolves yet after the Tibetan lineage^55^. Fossils of wolves have been present across most regions of China over the last 100,000 years, including 12,000-year-old remains from the Southern province of Jiangxi^56^. During this time, much of southern China was drier, with mixed broadleaf forests occupying where monsoon rain forests are now^57^. Thus, it is plausible that ancestry distinct from the modern-day Tibetan lineage could have existed within a glacial refugium in Southern China and has not been completely homogenized by expansion waves from northern Eurasian wolves. Incorporating ancient genomes from eastern China and the newly sequenced 35 kya Japanese wolf genome in future analyses would be needed to place this distinct ancestry into a spatiotemporal context during the Pleistocene^19^.

An unexpected result in our study was that ancestry from the Indian lineage is present in wolves across southwestern Asia. When and how the present-day population structure formed in southwestern Asia remains unclear, but our results offer some insights. Unlike eastern Asian wolves, which consistently form an early diverging lineage within Holarctic wolves, southwestern Asian wolves show differing phylogenetic relatedness across their genome—primarily clustering them within the Holarctic lineage or sister to the Indian wolf. However, the X chromosome was dominated by topologies reflecting the historical relationship with Indian wolves as the earliest diverging lineage, especially in regions of low (<0.2 cM/Mb) recombination. This historical relationship being most prevalent on the X chromosome is consistent with southwestern Asian wolves tracing their major genomic background back to the Holarctic lineage. Together, these results suggest ancient admixture with the Indian wolf contributed significantly to the genetic makeup of wolves in southwestern Asia. Ancient remains of wolves have been documented in southwestern Asia throughout the late Pleistocene, suggesting long-term continuity in wolf presence^56,58–60^. Genomes from these ancient wolf remains in southwestern Asia can directly test whether Indian ancestry was already present thousands of years ago, and whether there were shifts in Indian vs. Holarctic ancestry over the late Pleistocene.

Together, our work uses a combination of methodological approaches to better resolve the evolutionary history of a wide-ranging species. For many species during the late Pleistocene, their populations underwent distinct evolutionary trajectories of isolation and expansion in response to shifting climates. The process by which populations do not or do come together, and how often, is a key force in speciation and evolution. Our study’s findings of evolutionarily distinct wolf populations in southern regions may represent a common pattern in widespread species, as similarly documented in red foxes (*Vulpes vulpes*), Eurasian otters (*Lutra lutra*), and brown bears (*Ursus arctos*)^29,61,62^. While our study addresses gaps in our knowledge on wolves, many questions remain to be answered. For example, why some wolf populations seem to have been better connected with northern populations (southwestern Asia, eastern Asia) during the late Pleistocene, while others show little gene flow and prominent contact zones (Indian and Tibetan) is an open question. With many ancient and modern genomes now available, wolves are poised to serve as a valuable model to study how intra-population gene flow, interspecies hybridization, and isolation during the late Pleistocene have contributed to present-day species’ diversity.

### Conservation implications

Our study has several implications for the conservation of various wolf populations in Asia. For taxonomy, our study supports previous calls that a taxonomic revision is necessary for wolves in Asia^9,63,64^. While wolves from India to Türkiye are collectively described as *Canis lupus pallipes*, our study supports past work indicating that the Indian lineage is confined to India and parts of Pakistan^11,15,65^. In particular, our work using whole-genome sequences adds support for the proposed distribution of Indian and Tibetan wolves based on mitochondrial or limited nuclear genetic data^11,66,67^. Formally describing the Indian wolf as a distinct taxon from wolves in southwestern Asia is supported by these findings. Such recognition also highlights that Indian wolves are much more endangered than is currently recognized. Recently, it was estimated that ~2800 individuals occur in India and likely <300 individuals in Pakistan^68–70^. This population estimate and their ongoing decline placed the Indian wolf as Vulnerable under the IUCN Red List, indicating they are at high risk of extinction in the wild^70^. For the wolves in southwestern Asia that are currently considered “*Canis lupus pallipes*”, we find this distinct population may be geographically limited by the Bosphorus Strait in Türkiye and the Caucasus mountain range (Figs. 1a,b and 2a). Further work is necessary to understand the population genetic structure in the arid areas of southern Central Asia, such as the Karakum Desert in Turkmenistan and the Kyzylkum Desert in Uzbekistan. We suggest the morphological and genomic distinctiveness of wolves in southwestern Asia warrants subspecies status, in line with recent recommendations^9^.

In addition, our study clarifies the distribution and informs the conservation status of the Tibetan lineage. We found that Tibetan ancestry is highly restricted to the Tibetan plateau, with populations just adjacent (Pakistan, Tajikistan, and lowland India) having little Tibetan ancestry. With additional inference using whole-genome data in this study, we propose that the most western distributional extent of the Tibetan lineage is in the eastern Ladakh region of India^15,66,67^. Clarifying their distribution using genetics has already significantly impacted the conservation status of the Tibetan wolf, where they were recently assessed as Vulnerable on the IUCN Red List^71^. Our genetic diversity estimates for Indian and Tibetan wolves also emphasize their precarious status, as they have among the lowest genetic diversity of wolves included in our study. While their long-term small population size may have buffered against acquiring deleterious variation as suggested by our analyses of genetic load, they still face lower adaptive potential that may hinder their ability to adapt to future environmental changes^72^.

Our study underscores the importance of robustly describing wolves in parts of China. Wolves have been documented as far South as Guangxi in China, a province that borders Vietnam^56^ (Supplementary Fig. 1). In 1936, wolves were reported to exist in parts of Central and South China, but were already less common compared to Northern China^73^. A report in 2003 concluded wolves have largely been extirpated from regions of Guangxi where they once occurred in the 1990s, but they were possibly still present in Nanling National Nature Reserve^74^. In response to their decline in the country, the wolf was recently added in 2021 as a protected species under Class II of the List of Wildlife under Special State Protection of China. To the authors’ knowledge, we are not aware of published estimates of the population size and status, or a morphological description, of wolves in the Southern parts of China. Given their evolutionary distinctiveness, our work highlights the need for more research on their distribution, genetics, morphology, and population status to conserve what populations remain.

Overall, our study emphasizes that southern regions of Asia hold most of the evolutionarily distinct populations within grey wolves, and are also the most endangered. The Indian lineage numbers at ~2800 individuals in India and the Tibetan lineage at ~3000 individuals^68,71^. There are fewer than ~750 wolves left on the Arabian Peninsula, an evolutionarily unique but understudied population where only one genome has been sequenced^75^. It is possible that wolves in Southern China have already been extirpated. Wolves in these southern regions of Asia are impacted by numerous human-induced threats, especially for the remaining populations in Southern China and India. Our study highlights the need to prioritize these populations for conservation to preserve the full spectrum of extant wolf diversity.

## Methods

### Specimen information and processing

This study includes 20 new whole genomes from wolves from Pakistan (*n* = 8), Israel (*n* = 1), Lebanon (*n* = 1), Afghanistan (*n* = 1), Türkiye (*n* = 1), Ukraine (*n* = 1), Slovakia (*n* = 1), Kazakhstan (*n* = 3), and Russia (*n* = 3). Because wolves in Pakistan are listed as Appendix 1 under CITES, wolf samples from Pakistan were exported to the USA for sequencing under CITES permit number P-50/2020. Regional permits and permission to sequence samples for museum sample collection (Steinhardt Museum of Natural History, Natural History Museum of Denmark, Yekaterinburg Museum) were obtained for the rest of the wolf samples. For all wolf samples from Pakistan and Israel, we extracted DNA from these samples using the DNAeasy Blood and Tissue Kit (Qiagen) following the manufacturer’s protocol. Indexed libraries were constructed using NEBNext Ultra DNA Library Prep Kit for Illumina according to the manufacturer’s instructions, then cleaned and size selected using Agencourt AMPure XP beads (Beckman Coulter). The libraries were then sequenced on an Illumina NovaSeq 6000 S4 flowcell and paired-end at 150-bp (Illumina) at the UC Davis Genome Center. For the rest of the new wolf samples, they were extracted using the KingFisher Duo Prime Purification System (Thermofisher). Libraries were built using Beijing Genomics Institute (BGI) library protocols that were previously optimized^76,77^ using 10uM adaptors. These samples were sent to BGI Copenhagen for library build and sequenced on ⅛ lane each on DNBSEQ at paired-end 150. The Indian wolf from Maharashtra originated from a previous study and was resequenced to a deeper depth following protocols in Hennelly et al. 2021^11^. Lastly, we sequenced a wolf from Afghanistan that derived from a museum specimen from the Natural History Museum of Denmark (Museum ID: 4499). This specimen was collected in 1949, and we sampled the cartilage of the specimen. Following Gilbert et al. 2007^78^, the skin cartilage tissue was digested in a DDT, proteinase K-based buffer. The digest was purified following Dabney et al. 2013^79^ using a modified binding buffer^80^. The sample was built into a library using a modified single-tube protocol^76,77^. The sample was then sequenced on a BGI DNBSEQ. We constructed a dataset comprising an additional 95 previously published canid genomes, resulting in a total of 115 canid genomes in the study (Supplementary Data 1).

### Alignment, variant calling, and filtering

We used the Paleomix v.1.2.13.2. pipeline to align our raw reads to the dog reference genome Canfam3.1^81^. Specifically, we used the BWA mem algorithm for modern wolf samples and the BWA backtrack algorithm for the Afghan museum sample, and used the Genome Analysis Toolkit (GATK) indel realigner to generate an indel-realigned BAM file for each sample^82,83^. Variant calling was performed using the GATK version 4.2.5.0. First, we used GATK Haplotype Caller to perform variant calling on each sample, then used the resulting GVCF files to perform joint genotyping with all canid individuals combined using GenomicsDBImport and GenotypeGVCF in GATK. We obtained a set of high-quality single-nucleotide polymorphisms (SNPs) using the following filtering steps in GATK: QD < 2.0 | | SOR > 3.0 | | FS > 60.0 | | MQ < 40.0 || MQRankSum < −12.5||ReadPosRankSum < −8.0. Sites with a mean depth of >1800 for all individuals were removed to exclude paralogues from our dataset. Lastly, we removed indels from our dataset using SelectVariants in GATK (flag-select-type SNP).

Our resulting dataset included samples that varied in their average coverage (1.38x−43.46x). To account for lower coverage (<5×) samples and to fully use our dataset in the study, we used genotype likelihoods for the following analyses: principal component analysis with PCAngsd^84^, inferring individual admixture proportions with NGAdmix^85^, inferring genome-wide heterozygosity with ANGSD^86^, and estimating the inbreeding coefficient with NgsRelate^40^. We also used a per-site depth cutoff at more than ⅓ the average depth for higher coverage samples (>15x) for the PSMC and ROH analyses, a minimum per-side depth of 10x for our genetic load and fastsimcoal2 analysis. Due to their low coverage, we excluded our wolf from the Afghanistan sample that was derived from a museum specimen and the Ethiopian wolf in our genotype calling with GATK, and only used this sample for analysis using genotype likelihoods.

### Principal component analysis and individual admixture proportions

To assess the genome-wide genetic structure of the autosomes, we conducted a principal component analysis (PCA) using PCangsd^85^ with our total set of Eurasian grey wolf genomes (*n* = 98). We inferred the PCA using genotype likelihoods from ANGSD, in which we used the SAMtools model (-GL 1) and filtered the dataset by including only properly paired reads (-only_proper_paired 1) from autosomal bam files, excluded reads with excess of mismatches (-C 50) or mapping quality lower than 20 (-minMapQ20), removed transitions (-noTrans 1), and keep bases with a sequence quality above 20 (-minQ 20)^86^. We also retained SNPs that were present in at least 90% of individuals present (-minInd 88), resulting in ~10 million autosomal SNPs. We obtained the covariance matrix from PCAngsd and used the R function *eigen* to infer the PCA.

To estimate individual ancestry proportions, we inferred genotype likelihoods using ANGSD and ran NGSadmix^85^ that included (1) grey wolves, dogs, and other species for *K* = 2 to *K* = 8 clusters and (2) only grey wolves for *K* = 2 to *K* = 6 clusters. For the first run, we included grey wolves from Eurasia and North America (*n* = 101), dogs (*n* = 4), a golden jackal (*n* = 1), coyotes (*n* = 2), African wolves (*n* = 4), an Ethiopian wolf (*n* = 1), and a dhole (*n* = 1) (Supplementary Data 1). For the second run, we used only grey wolves from Eurasia (*n* = 98) and North America (*n* = 3). We used the same criteria in ANGSD to infer the PCA, and retained SNPs that were present in at least 90% of individuals in both runs. This resulted in 10,626,992 SNPs and 10,907,278 SNPs after filtering for the first and second run, respectively. We calculated the cross-validation score (CV) using the command –cv, in which the highest score indicates the value of *K* for the model that has the best predictive accuracy. Because of the hierarchical structure of natural populations, we examined in detail multiple levels of *K*, where each can provide information on subpopulation structure. We present all levels of *K* and cross-validation scores in the supplemental materials, but we highlight *K* = 6 because it provides higher resolution of the subpopulation structure and admixture proportions corresponding to interpopulation and interspecific admixture. Because of higher dog ancestry detected with NGSAdmix in the two Iberian wolf samples, we removed the two Iberian wolves from the heterozygosity analysis, ROH analysis, PSMC, and timing of inbreeding analysis.

### Assess barriers to gene flow for wolves across Eurasia

We inferred the estimated effective migration surface (EEMS) to assess spatial variation in rates of gene flow among grey wolf populations in Eurasia^20^. The FEEMS approach utilizes a stepping-stone model to calculate genetic dissimilarities between individuals based on spatial and genetic data. These genetic dissimilarities can be visualized to illustrate departures from strict isolation by distance, thereby detecting regions of high (i.e., corridors) and low (i.e., barriers to) gene flow. For the FEEMS, we only used our dataset of 97 Eurasian wolves, where we removed the low coverage wolf from Afghanistan, filtered the dataset using VCFtools version 1.16 in which we kept sites with a minimum allele count of 3 (--mac 3), all of individuals represented at a site (--geno 0), and pruned sites in high linkage disequilibrium by removing each SNP with a *r*^2^ value of greater than 0.5 (--indep-pairwise 50 10 0.5)^87^. This resulted in ~8.7 million autosomal SNPs across our Eurasian wolves. For gathering the geographic data for each wolf sample, we had some grey wolf samples that only had location information as the country or province within a country. In these cases, we took the center coordinate within that country or province.

To run FEEMs, we first constructed a dense spatial grid using an edge width of 5 that covered the entire Eurasian continent. We initially performed a random initialization over the map to fix the estimate of the residual variance. We selected the tuning parameter, lambda, using leave-one-out cross-validation using 20 folds, where the lambda with the lowest cross-validation was selected. Our cross-validation test found a lambda value of 100 to be the optimal value of lambda (Supplemental Fig. 22).

### Phylogenomic relationships

We constructed the autosomal and X chromosome phylogeny that included all samples in our study except dogs and the Ethiopian wolf. We inferred the X chromosome phylogeny due to its differences in inheritance patterns and Ne compared to the autosome, providing additional insight into the evolutionary history of grey wolves. We filtered the autosomal and X chromosome VCF to exclude indels, keep sites with at least a minimum quality of 30 (--minQ30), keep only biallelic sites, and keep sites that had at least 90% individuals called (-max-missing 0.9). For the autosomal tree, we extracted 1000 randomly selected regions with a length of 20 kb that only included variant sites using bedtools random^88^. We converted each VCF file to a phylip file format using vcf2phylip.py (https://github.com/edgardomortiz/vcf2phylip). Next, we inferred a maximum-likelihood phylogenetic tree for each 20 kb region using IQ-Tree 1.6.12, where we estimated the best model using ModelFinder and used 1,000 ultra-fast bootstraps to infer each tree^89,90^.

To quantify what proportion of these randomly selected 1000 20 kb windows fell into different recombination rate regions, we downloaded the recombination map of the domestic dog from Auton et al. 2013^91^ to use in our recombination rate analyses. We averaged the recombination rates within 100-SNP windows across the autosomes and X chromosome, as generated and detailed in a previous study^11^. We selected genomic regions that had an average recombination rate within each 100-SNP window of below 0.2 cM/Mb, considered as low recombination rate, 0.2 cM/Mb to 2 cM/Mb, considered as medium recombination rate, and above 2 cM/Mb, considered as high recombination rate. We used these positions of each 100-SNP window to create a bed file for low recombination, medium recombination, and high recombination regions. We then used these bed files to extract these genomic regions from our VCF using bedtools intersect. For our randomly selected 1000 20 kb regions, ~15.5% of the genomic regions fell within low recombination regions (<0.2 cM/Mb), ~61.3% fell within medium recombination regions (0.2 cM/Mb-2cM/Mb), and ~23.1% fell within high recombination regions (2 cM/Mb). This proportion is similar to the overall proportion across the autosomes that fall within low (<0.2 cM/Mb), medium (0.2–2 cM/Mb), and high (>2 cM/Mb) recombination categories, which were ~18.8%, 62,3% and 18.2% of the total 100-SNP windows across the autosomes, respectively, as reported in Hennelly et al. 2021. The inferred phylogenetic trees were used as input into ASTRAL 5.7.8, which inferred a species tree from a set of gene trees while taking into account gene tree discordance (Zhang et al. 2018, p. 90). For the X chromosome, we converted the filtered VCF file to a phylip file format using vcf2phylip.py and then inferred a maximum-likelihood phylogenetic tree using IQ-Tree 1.6.12, where we estimated the best model using ModelFinder and used 1000 ultra-fast bootstraps^89,90^.

In addition, we inferred phylogenetic trees of the X chromosome using only the low recombination regions across the X chromosome. This is because low recombination regions are more likely to retain the historical relationship due to higher selection against introgressed ancestry and higher lineage sorting^24–26^. We inferred the X chromosome phylogeny using only low recombination (<0.2 cm/cM), using all individuals in the study except dogs and the Ethiopian wolf. We excluded the Ethiopian wolf from our analyses due to its low coverage. In addition, we inferred two more X chromosome phylogenies using only low recombination regions that excluded different wolf populations to investigate how phylogenetic relationships vary depending on what populations are included in the analysis. This is because the topology of genome-wide phylogenies can be influenced by many evolutionary processes, including admixture between different populations and/or species. Inferring phylogenetic trees with different sets of individuals can help reveal phylogenetic discordance among populations and understand the evolutionary processes underlying these discordances.

For these two X chromosome phylogenies using only low recombination regions, we inferred phylogenies where they included (1) all samples in our study excluding dogs, the Ethiopian wolf, wolves from Pakistan and Ladakh, and wolves from southwestern Asia and (2) all samples in our study excluding dogs, the Ethiopian wolf, wolves from Pakistan and Ladakh, and Indian wolves. We hypothesize that if Indian and southwestern Asian wolves are sister lineages as found in the autosomal phylogeny, then the removal of one population will not influence the topological relationship of the other due to their overall shared evolutionary histories. If the sister relationship between Indian and southwestern Asian wolves is due to minor ancestry via gene flow within southwestern Asian wolves, removing southwestern Asian wolves will result in the Indian lineage being the earliest diverging lineage. This is due to the Indian lineage no longer experiencing affinity towards the minor Indian ancestry present in southwestern Asian wolves.

For inferring the three sets of X chromosome phylogenies using only low recombination regions, we filtered all VCF files for sites with a minimum quality of 30 (-minQ 30), only biallelic sites, and at least 90% individuals present at each site (--max-missing 0.9. We then used our bed file containing coordinates to low recombination regions of the X chromosome to extract these genomic regions from our VCF using bedtools intersect. We converted the VCF containing all low recombination regions to a phylip file format using vcf2phylip.py (https://github.com/edgardomortiz/vcf2phylip). We then ran IQ-Tree 1.6.12 with estimating the best model using ModelFinder and used 1000 ultra-fast bootstraps to infer each tree^89,90^.

### Assessing genome-wide gene admixture

To test for gene flow among various canid lineages and species, we performed *D*-statistic analyses using ADMIXTOOLS^22^. We filtered our dataset to exclude missing data (--max-missing 1), kept biallelic sites, and applied a minimum quality filter of 30 (--minQ30), along with the filters we applied using GATK. We converted plink files into eigenstrat format using the convertf script within ADMIXTOOLS. We calculated the *D*-statistic and *Z*-score for modern populations for four different topologies in the format (((P1, P2),P3),P4): (((Norwegian wolf MW005, X wolf); Indian wolf BH123); Andean fox), (((Norwegian wolf MW005, X wolf); Tibetan wolf TI32); Andean fox), (((Norwegian wolf MW005, X wolf); African wolf); Andean fox), (((Norwegian wolf MW005, X wolf); golden jackal); Andean fox). We selected the wolf from Norway as P1 because this population does not overlap with any closely related wild canid species, and low rates of wolf-dog hybridization in Scandinavia^92^. The *D*-statistic value will be negative for an excess of shared derived alleles between P2 and P3, and/or P1 and P4. Alternatively, the *D*-statistic will be positive for an excess of shared derived variants between P2 and P4, and/or P3 and P1^93^. For African wolves, we used multiple individuals as a population for P3, which consisted of all four of our African wolf samples from Kenya, Ethiopia, Morocco, and Algeria. For the golden jackal, we used the golden jackal from Syria, which does not show any signatures of recent admixture with any other canid species according to the individual admixture proportions (Supplemental Fig. 2).

We also quantified the frequency of different topologies in genomic windows across the genome using Twisst^94^. To prepare the dataset for the Twisst analysis, we first phased the filtered dataset that contained no indels, biallelic sites with a minimum quality of 30, and that were present in at least 90% of individuals using Shapeit v2.r904^95^. We used the dog genetic map from Auton et al. 2013^91^ as input and a window size of 0.5 Mb. We converted the phased VCF to a geno file using the parseVCF.py script within https://github.com/simonhmartin/genomics_general. We then estimated phylogenies in 100-site windows using phyml with a general time reversible (GTR) substitution model with phyml_sliding_windows.py within https://github.com/simonhmartin/genomics_general. We summarized the relative prevalence of topologies using the twisst.py script. The set of topologies we summarized consisted of four population groups with Andean fox as an outgroup, focused on wolves in Central Asia and Eastern Asia, and five population groups with Andean fox as an outgroup, focused on southwestern Asian wolves (Supplementary Table 1). These result in 15 possible topologies for the four population groups, and 105 possible topologies using five population groups. We also quantified the frequency with which topologies occurred in windows of low (<0.2 cM/Mb), medium (0.2–2 cM/Mb), and high (>2 Mb/Mb) recombination rate regions. To do this, we averaged recombination rates within 100-SNP windows inferred in Hennelly et al. 2021—11 across the autosomes and X chromosome datasets. We then extracted 100-SNP windows inferred using twisst that overlapped within the low, medium, and high recombination regions for the autosomes and X chromosome using bedtools intersect. We averaged the topology weightings of each topology found within each recombination rate category for the autosomes and X chromosome.

### Demographic modeling with fastsimcoal2

In order to better understand the origin of southwestern Asian wolves, we compared three alternative demographic models and inferred demographic parameters under the FASTSIMCOAL2^37^, a composite-likelihood method that estimates historical introgression, divergence times, and population topologies from the site frequency spectrum using coalescent simulations.

We compared three demographic models (Supplemental Fig. 16) to better assess the demographic history of southwestern Asian wolves. The included three populations representing India, southwestern Asia and central Asia samples, and were defined as (1) a divergent model with a strictly bifurcating topology separating Indian and Holarctic Asian lineages and latter southwestern Asia from central Asia populations, (2) an introgression model with the same strictly bifurcating topology followed by an introgression event from the Indian to the southwestern Asian population (3) an hybridization model, where southwestern Asian population was originated by hybridization between Indian and central Asian populations. Input files, including template and parameter estimation files for the three models, are available at Supplemental Fig. 25. A summary of all defined parameters and their search ranges is given in Supplementary Table 2.

We performed genotype calling using BCFtools, specifically keeping monomorphic sites^96^. We filtered our VCF containing all variants and monomorphic sites by the following parameters, keeping only those loci with >10× coverage, and >50% missing data, and removing sites SNPs violating Hardy–Weinberg equilibrium for heterozygous excess (*p* < 0.01). We used the canFam 3.1 domestic dog assembly as the reference, resulting in the reference being more closely related to Holarctic wolves than to Indian wolves. Thus, we could not determine the ancestral state of each allele and therefore used the minor allele frequency spectrum (folded SFS). The mutation rate was set to 4.5 × 10^−9^ based on de novo mutations in a pedigree of wolves^97^.

To generate the 3D-SFS while ensuring no missing data and while considering local linkage disequilibrium (LD) patterns, we down-sampled individuals. Using the recombination map of the dog genome^91^, which estimates a recombination rate of 1.34 cM/Mb, we divided the genome into independent and unlinked blocks of 2 Mb. This approach ensures that sites within blocks are linked, while those between different blocks are unlinked. Within each block, we kept only SNPs without missing data by sampling three individuals from the Indian population, six from the southwestern Asia population, and nine from the central Asian population, ensuring no missing data for each sampled individual in that block. In total, we therefore retained 2,771,059 SNPs and 456,437 monomorphic sites, which we used to generate the joint 3D-SFS using the (folded) minor allele frequency spectrum.

FASTSIMCOAL2 estimates a composite likelihood, assuming independence between sites. Consequently, while the maximized likelihood converges to the correct parameter values, allowing for parameter estimation even from linked SNPs, the estimated likelihood is more accurate when derived from a set of unlinked loci^37^. Therefore, to compare the likelihoods obtained under different models, we need to recalculate the likelihood using unlinked SNPs. We used the parameter estimates from all retained SNPs to recalculate the likelihoods for each model. This recalculation was based on a 3D-SFS derived from a set of potentially independent SNPs, with one SNP selected per block, resulting in 1069 SNPs. We used this newly computed likelihood to apply the Akaike Information Criterion (AIC) to compare the three models. For this, we calculated delta AIC according to Excoffier et al. (2013)^37^, which represents the difference between the AIC value of each model and the minimum AIC value among all models. Further, we thoroughly examined the parameter values that optimized the likelihood for each model. Specifically, we compared divergence times between Holarctic and Indian lineages, the effective population sizes of each population, and the admixture proportions estimated under each model. This analysis aimed to determine whether the parameter estimates that optimized each model were consistent with the most likely model as indicated by the AIC. In addition, to evaluate whether the best-selected model could accurately reproduce the observed data, we conducted a visual examination comparing the expected and observed marginal one-dimensional SFS, as well as various two-dimensional SFS pairs.

### Long-term demographic history with PSMC

To infer the historical demography of wolves in Eurasia, we used the pairwise sequential Markovian coalescent (PSMC)^38^. PSMC uses a coalescent approach to estimate the history of change in effective population sizes over time. We only included the autosomal sequences of each grey wolf individual. We converted each bam file to a fasta-like consensus sequence by first using the mpileup command with SAMtools and subsequently using BCFtools view –c to call variants and vcfultils.pl vcf2fq to convert the vcf file to fastq format (Danecek et al. 2021, p. 95). We excluded any reads that were <20 for minimum mapping quality and minimum base quality (-q 20 -Q 20) and excluded reads with excessive mismatches (-C 50). We also removed sites with more than double or less than a third of the average depth of coverage for each sample. We tested different combinations of parameters to infer the PSMC, which were “psmc -N25 -t15 -r5 -p 4 + 25*2 + 4 + 6”, “psmc -N25 -t15 -r5 -p 2 + 2 + 25*2 + 4 + 6”, and “psmc -N25 -t15 -r5 -p 1 + 1 + 1 + 1 + 25*2 + 4 + 6” following previous studies on grey wolves and recent recommendations for inferring PSMC trajectories^97^. Some samples showed a false peak when using the parameter “psmc -N25 -t15 -r5 -p 4 + 25*2 + 4 + 6”; therefore, we used the parameter “psmc -N25 -t15 -r5 -p 1 + 1 + 1 + 1 + 25*2 + 4 + 6” for our final analyses (Supplemental Fig. 23).

To account for the limitations of our lower-coverage genomes (15–20×), which may miss heterozygous sites due to insufficient depth, we estimated the false negative rate (FNR) by downsampling a high-coverage grey wolf genome from the same geographic region to match the specific depth of the lower-coverage genome. To determine the best FNR, we visually compared PSMC plots from a high coverage (>20×) grey wolf from the same region with plots from its downsampled versions corrected using various FNR values (Supplemental Fig. 24). We chose the FNR that gave the closest match and applied it to the low coverage grey wolf genomes to infer their demographic history. For this, we used a mutation rate of 4.5 × 10^−9^^98^ and a generation time of 4.4 years^99^.

### Genetic diversity and runs of homozygosity

We calculated the genome-wide heterozygosity for each wolf individual above 5x using ANGSD^86^. We first estimated the folded site allele frequency with the realSFS program in ANGSD (-doSaf) using genotype likelihoods with the SAMtools model (-GL 1) and using the Andean fox genome to determine ancestral state. We filtered each individual bam file to keep only autosomal chromosomes, only properly paired reads (-only_proper_paired 1), excluded reads with excess of mismatches (-C 50), excluded reads with a mapping quality (-minMapQ 20), excluded reads with a depth less than 4 (-setMinDepth 4), and kept bases with a sequence quality above 20 (-minQ 20). To calculate the genome-wide heterozygosity, we divided the number of heterozygous sites by the total number of sites for each sample.

We inferred runs of homozygosity (ROH) using BCFtools/RoH^41^. Along with the GATK filtering criteria, we included only biallelic SNPs that had all individuals present per site (--max-missing 1, --min-alleles 2, --max-alleles 2). We also included SNPs that had a depth of more than one-third and less than double the average depth of coverage for each sample, where we chose samples that were >15×. For the BCFtools/RoH analysis, we fixed the alternative allele frequency to 0.4 (-AFdflt 0.4; default setting) and used the dog recombination map from Auton et al. 2013^91^ to account for recombination hotspots (--genetic-map). We kept ROHs that had a quality score of at least 80 and excluded ROHs that were below 100 kb in length in our analysis. We inferred the timing of inbreeding by the equation *g* = 100(2*rL*), where *g* corresponds to generation time, *L* to the length of ROH in Mb, and *r* as the average genome-wide recombination rate (1.34 cM/Mb, averaged from the recombination rates in Auton et al. 2013^91^). We note this method does not account for recombination rate variation across the genome, and therefore, our inbreeding timing dates should be considered as estimates.

### Inbreeding coefficient

We inferred the per-individual inbreeding coefficient (*F*_ind_) using genotype likelihoods, where the inbreeding coefficient is the probability of identity by descent. We used ANGSD to infer genotype likelihoods and allele frequencies for samples above 4x coverage with the following parameters: -GL 2 -checkBamHeaders 0 -trim 0 -C 50 -baq 1 -skipTriallelic 1 -minMapQ 20 -minQ 20 -minInd 88 -uniqueOnly 1 -remove-bads 1 -noTrans 1 -doGlf 3 -doMajorMinor 1 -doMaf 1 -minMaf 0.0001 -SNP_pval 1e-6. We then used NgsRelate v2 with option -F1 to infer the inbreeding coefficients for each Eurasian wolf individual^40^.

### Genetic load

We selected 14 individuals with >20× sequencing coverage from India (*n* = 2), the Tibetan plateau (*n* = 2), southwestern Asia (*n* = 4), Central Asia (*n* = 4), and the Mexican wolf (*n* = 1). We selected these wolves to assess the genetic load of Indian and Tibetan wolves, in which we compared Indian and Tibetan wolf genetic load with two adjacent, genetically diverse populations, and a Mexican wolf individual with previously documented very low genetic diversity. We used SnpEff to annotate a VCF, including only biallelic non-indel sites with no missing data, a minimum depth of 10, a maximum depth of 60, a minimum allele count of 2, and a minimum sequence quality (--minQ) of 30.

To polarize our dataset, we followed the method by Smeds et al. (2022)^49^ to map two outgroups to the dog reference genome at only sites with a minimum depth of 4 at both outgroup and included nonvariant sites. We used Dhole (*Cuon alpinus*) and Andean fox (*Lycalopex culpaeus*) as our outgroups. We then performed pseudo-haploidization of the outgroup genomes and kept only sites in which the allele agreed in both outgroups (Smeds et al. 2022). We used this to add the ancestral allele information to our annotated VCF, and assumed that the derived allele was the deleterious allele^49^.

We counted the number of derived alleles and whether they were in a homozygous or heterozygous state for variants predicted by SnpEff to have high, moderate, and low impact on protein function. High impact is predicted to cause loss of function or protein truncation (stop gain, frameshift mutation); moderate impact is predicted to change protein effectiveness (missense variant), and low impact is assumed harmless, such as a synonymous variant. We also calculated the following metrics: total load (the total number of derived alleles classified as high or moderate impact), realized load (homozygous state of derived alleles in high or moderate impact), and masked load (heterozygous state of derived alleles in high or moderate impact).

To investigate evidence of differential loss of derived variants for different types of mutations, we assessed the relative amounts of mutational categories given by SnpEff. These mutational categories are: Loss of Function (LOF) mutations that imply a high predicted impact on protein structure, missense mutations (moderate impact), synonymous mutations (low impact), and “modifier” mutations found in non-coding regions (no impact). We calculated the count of derived alleles and the number of heterozygous and homozygous derived allele calls in each of the four mutational categories for each wolf individual. To account for differences in overall derived allele counts among individuals, we calculated the average derived allele count per 100,000 derived neutral alleles for total mutational load, LOF, missense, and synonymous mutations. We considered the modifier category as neutral derived alleles. Specifically, we calculated mutational load as: (total derived alleles in LOF and missense/total derived alleles in modifier category) multiplied by 100,000.

### Reporting summary

Further information on research design is available in the Nature Portfolio Reporting Summary linked to this article.

## Supplementary information

Supplementary Information

Description of Additional Supplementary Materials

Supplementary Data 1

Reporting Summary

## Data Availability

All raw reads are publicly available on the National Center for Biotechnology Information Sequence Read Archive under Project ID PRJNA1285574, with accession numbers SRR35174885- SRR34347608. Results and tables of the analyses can be found on GitHub: https://github.com/hennelly/Asia_wide_wolf_genomics.

## Code availability

The bioinformatic scripts can be found on GitHub: https://github.com/hennelly/Asia_wide_wolf_genomics.
