# Publisher Correction: Continent-wide view of genomic diversity and divergence in the wolves of Asia

**DOI:** 10.1038/s42003-026-10138-7

**Published:** 2026-05-18

**Authors:** Lauren M. Hennelly, Bárbara R. Parreira, Ash Noble, Camilla H. Scharff-Olsen, M. Çisel Kemahlı Aytekin, Çağan H. Şekercioğlu, Pavel Kosintsev, Ladislav Paule, Pavel Hulva, Hans K. Stenøien, Bilal Habib, Hira Fatima, Ghulam Sarwar, Samara P. El-Haddad, Alexandra Youssef, Frank Hailer, Xin Sun, Nuno Filipes Gomes Martins, M. Thomas P. Gilbert, Benjamin N. Sacks, Mikkel-Holger S. Sinding, Shyam Gopalakrishnan

**Affiliations:** 1 Section for Evolutionary Hologenomics, Globe Institute, University of Copenhagen, Copenhagen, Denmark; 2 Center for Conservation Genomics, Smithsonian’s National Zoo and Conservation Biology Institute, Washington, DC USA; 3 Mammalian Ecology and Conservation Unit, Veterinary Genetics Laboratory, Department of Population Health and Reproduction, School of Veterinary Medicine, University of California, Davis, CA USA; 4 Department of BioSciences, Rice University, Houston, TX USA; 5 Organisms and Environment, School of Biosciences, Cardiff University, Cardiff, UK; 6 Department of Molecular Biology and Genetics, Koç University, Istanbul, Türkiye; 7 School of Biological Sciences, University of Utah, Salt Lake City, UT USA; 8 Kuzey Doğa Society, Kars, Türkiye; 9 Ural Federal University, Yekaterinburg, Russia; 10 Institute of Plant and Animal Ecology, Ural Branch of Russian Academy of Science, Yekaterinburg, Russia; 11 Technical University in Zvolen, Zvolen, Slovakia; 12 Department of Zoology, Charles University, Prague, Czechia; 13 Department of Biology and Ecology, University of Ostrava, Ostrava, Czechia; 14 NTNU University Museum, Norwegian University of Science and Technology, Trondheim, Norway; 15 Wildlife Institute of India, Dehradun, India; 16 University of Education – Attock Campus, Attock, Pakistan; 17 University of the Punjab, Lahore, Pakistan; 18 Lebanese Wildlife, Jitta, El Metn Lebanon; 19 Cardiff University-Institute of Zoology Joint Laboratory for Biocomplexity Research (CIBR), Beijing, China; 20 Department of Biology, University of Copenhagen, Copenhagen, Denmark

**Keywords:** Evolutionary genetics, Ecological genetics, Phylogenetics

Correction to: *Communications Biology* 10.1038/s42003-025-09379-9, published online 24 December 2025

In the original version of the article, Figure 5 appeared incorrectly in the HTML version. The article has now been corrected.

