# Supplementary material for: Continent-wide view of genomic diversity and divergence in the wolves of Asia
Source: Commun Biol. 2025 Dec 24;9:330. doi: 10.1038/s42003-025-09379-9 (PMC12953585; doi:10.1038/s42003-025-09379-9)
Supplement: Supplementary file 1 — Supplementary Information [file 42003_2025_9379_MOESM1_ESM.pdf]

**Supplementary Information for**
**"Continent-wide view of genomic diversity and divergence in the wolves of Asia"**

**Supplementary Figure 1.** The estimated distribution of the three major wolf lineages – the Indian, Tibetan, and Holarctic lineage
**Supplementary Figure 2.** Individual admixture proportions using K=2 to K=8 populations for 114 individuals consisting of various canid species, wolves, and dogs
**Supplementary Figure 3.** Individual admixture proportions using K=2 to K=6 populations for 98 gray wolves from Eurasia.
**Supplementary Figure 4**. Wolf sample location on a close-up of Central and South Asia with each sample's individual admixture proportions at K=6 for 98 wolves across Eurasia
**Supplementary Figure 5.** D statistic values to assess derived allele sharing between each wolf individual in Eurasia (X) with the Tibetan wolf with the topology: (Graywolf$_{Norway}$, X), Tibetan wolf–TI32), Andeanfox).
**Supplementary Figure 6.** D statistic values to assess derived allele sharing between each wolf individual X with the Indian wolf with the topology: (Graywolf$_{Norway}$, X), Indian wolf BH123), Andeanfox).
**Supplementary Figure 7.** D statistic values to assess derived allele sharing between each wolf individual (X) with African wolves with the topology: (Graywolf$_{Norway}$, X), African wolf), Andeanfox).
**Supplementary Figure 8.** D statistic values to assess derived allele sharing between each wolf individual (X) with golden jackals with the topology: (Graywolf$_{Norway}$, X), Golden jackal), Andeanfox).
**Supplementary Figure 9.** Derived allele sharing between each wolf individual in Eurasia (circles) with the Tibetan wolf with the topology: (Graywolf$_{Norway}$, X), Tibetan wolf–TI32), Andeanfox).
**Supplementary Figure 10.** Fully labeled autosomal phylogeny inferred a multispecies coalescent tree in ASTRAL using 1,000 randomly-selected regions with a length of 20kb across the autosomes using 107 individuals
**Supplementary Figure 11**. Fully labeled phylogeny using only low recombination regions (<0.2cM/Mb) across the autosomes, consisting of 3,625,481 SNPs
**Supplementary Figure 12**. Fully labeled phylogenetic tree of the X chromosome using 513,963 SNPs found in only the low recombination regions (<0.2cM/Mb) of 107 canid individuals
**Supplementary Figure 13**. Fully labeled phylogenetic tree of the X chromosome using 2,761,668 SNPs of 107 canid individuals
**Supplementary Figure 14. (A)** Fifteen possible topologies using the Andean fox (AF) as an outgroup and four different gray wolf populations: Tibetan (Tb), Central Russia (Rus), European (Eur), and X, which denotes either east Asian wolves or high altitude Central Asian wolves
**Supplementary Figure 15.** Derived allele sharing between each wolf individual in Eurasia (circles) with the Indian wolf with the topology: (Graywolf$_{Norway}$, X), Indian wolf–BH123), Andeanfox).
**Supplementary Figure 16.** Three alternative scenarios for the origin of the Southwest Asian population tested in fastsimcoal2 (Excoffier et al. 2021).

**Supplementary Figure 17**. (A, B) Maximum likelihood phylogeny of wild canids inferred with IQ-Tree 1.6.12 using only low recombination (<0.2cM/Mb) regions of the X chromosome.
**Supplementary Figure 18**. Estimated inbreeding coefficients ($F_{Ind}$) using genotype likelihoods with NgsRelate for 96 individuals across seven wolf populations in Eurasia.
**Supplementary Figure 19.** Timing of inbreeding estimated from the length of ROH blocks across the autosomes of 40 wolves
**Supplementary Figure 20.** Number of heterozygous and homozygous counts for the derived allele for High impact, Medium impact, and Low impact categories
**Supplementary Figure 21.** Total genetic load (the total number of derived alleles; homozygous derived alleles counted twice, heterozygous derived alleles once), realized load (homozygous state of derived alleles), and masked load (heterozygous state of derived alleles) for each impact category (High, Moderate, Low) for five selected wolf populations of wolves
**Supplementary Figure 22**. Cross validation results by using leave-one-out cross validation to select the optimal value of lambda, the smoothing parameter.
**Supplementary Figure 23.** PSMC plots for three wolf genomes while varying the atomic time intervals
**Supplementary Figure 24**. Down-sampling of genomes to select the false negative rates (FNR) for low-depth corrections of pairwise sequentially Markovian coalescent (PSMC) demographic trajectories
**Supplementary Figure 25.** Est and Tpl input files for fastsimcoal2 for each of the three models we tested.
**Models [1-3].**
**Supplementary Table 1**. Estimated likelihoods for the three demographic models
**Supplementary Table 2**. Demographic parameters and their search ranges as used in the FASTSIMCOAL2 analyses.

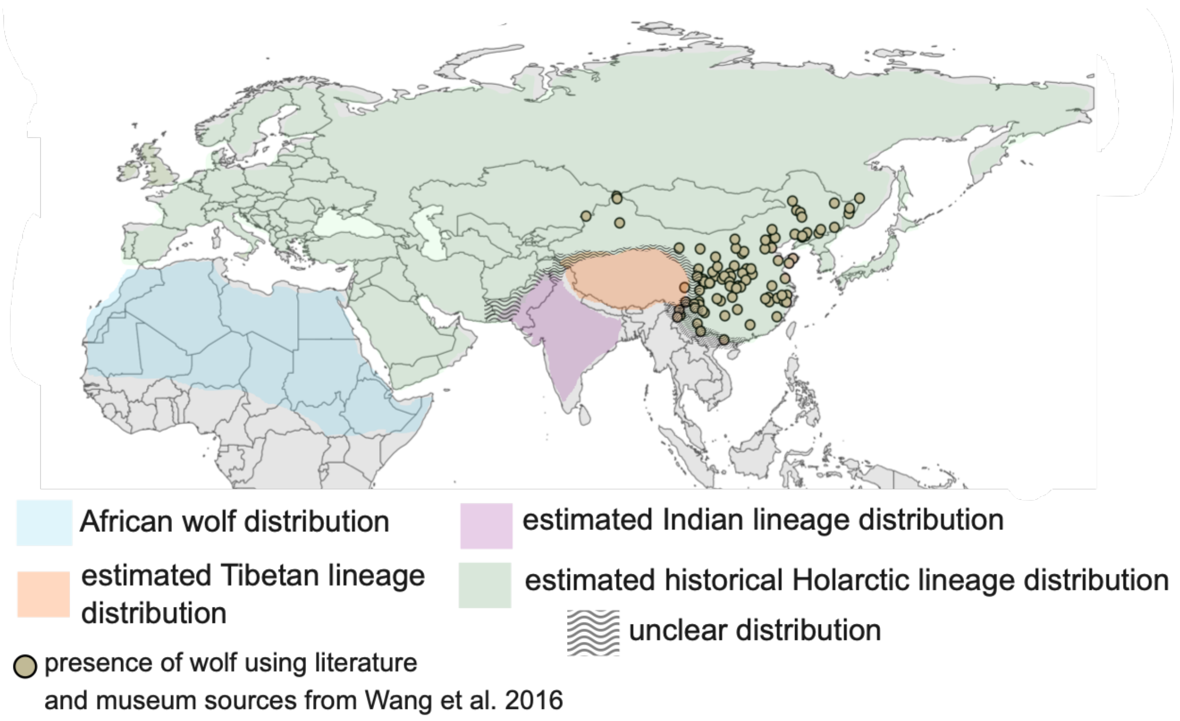


**Figure S1.** The estimated distribution of the three major wolf lineages – the Indian, Tibetan, and Holarctic lineage. The estimated distribution is based on previous work on geographic distribution of mitochondrial haplotypes for the three wolf lineages (Ersmark et al. 2016, Werhahn et al. 2023, Hennelly et al. 2023, Werhahn et al. 2020, Hoffmann and Atickem 2019). We plotted the estimated historical (pre-1500) distribution of the Holarctic lineage. Previous studies found concordance between mitochondrial haplotype and nuclear genomic ancestry for the Indian, Tibetan, and Holarctic wolf lineages (Werhahn et al. 2020, Wang et al. 2020, Hennelly et al. 2021, Hennelly et al. 2023). Wavy lines indicate unclear distribution due to lack of data. Circles indicate locations of wolf presence based on literature sources and museum specimens within Table 1 and Table 2 of Wang et al. 2016. Records of wolves are found throughout China, including southern regions bordering Vietnam. Wavy lines indicate uncertain distributional edges of the Indian and Tibetan lineages, as well as the southern distribution extent of the gray wolf in China (Wang et al. 2019).

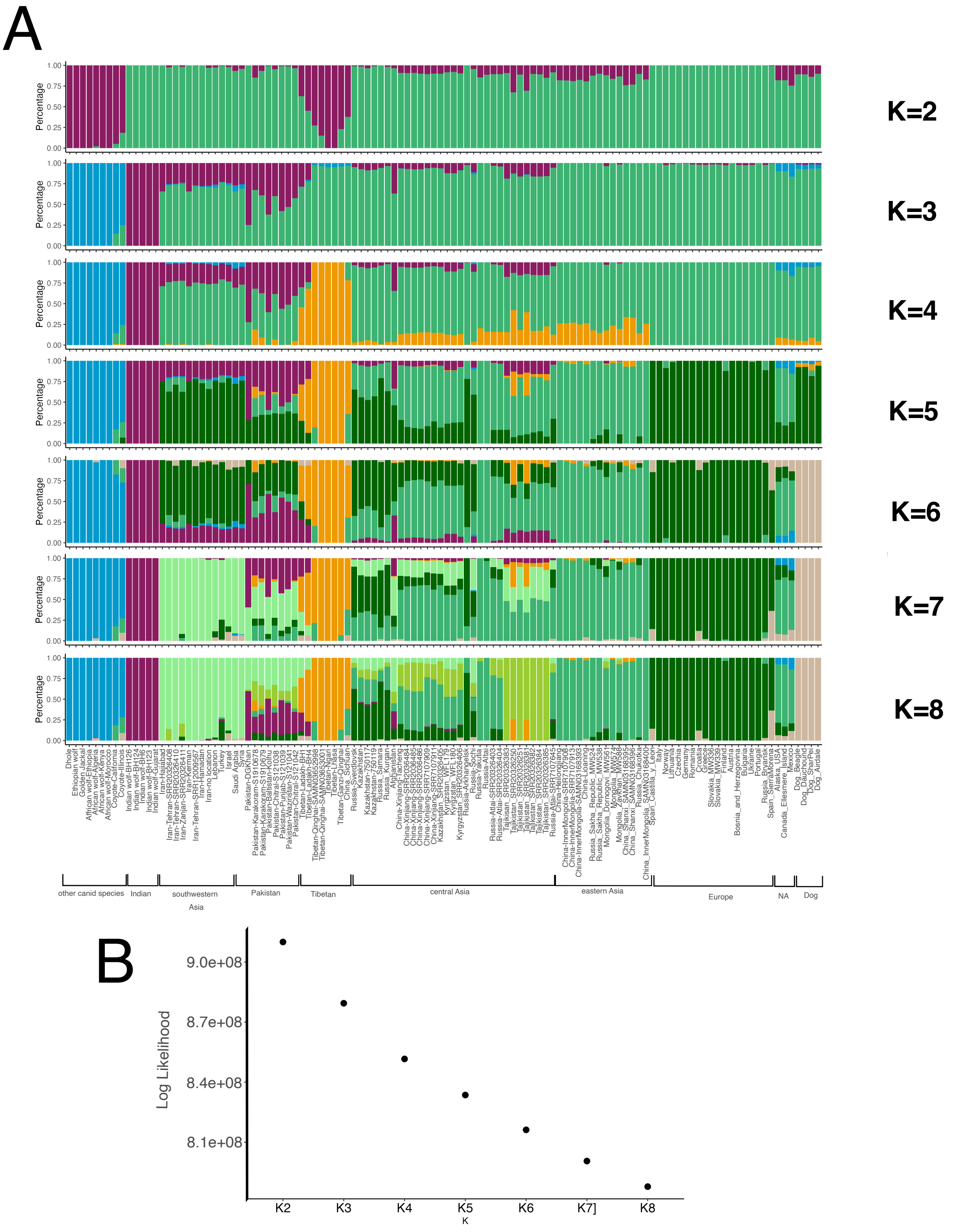


**Figure S2.** Individual admixture proportions using K=2 to K=8 populations for 114 individuals consisting of 78 wolves from Eurasia, three wolves from North America, 4 dogs,

1 Dhole, 1 Ethiopian wolf, 1 golden jackal, 4 African wolves, and 2 coyotes. We used 10,907,278 SNPs inferred using genotype likelihoods with ANGSD, which were used to estimate the individual admixture proportions through NGSAdmix (Skotte et al. 2013, Korneliussen et al. 2014). **(A)** Each bar represents an individual and colors within each bar represent the estimated ancestry belonging to a specific ancestry. **(B)** Log likelihoods of each run in NGSAdmix for K=2 to K=8.

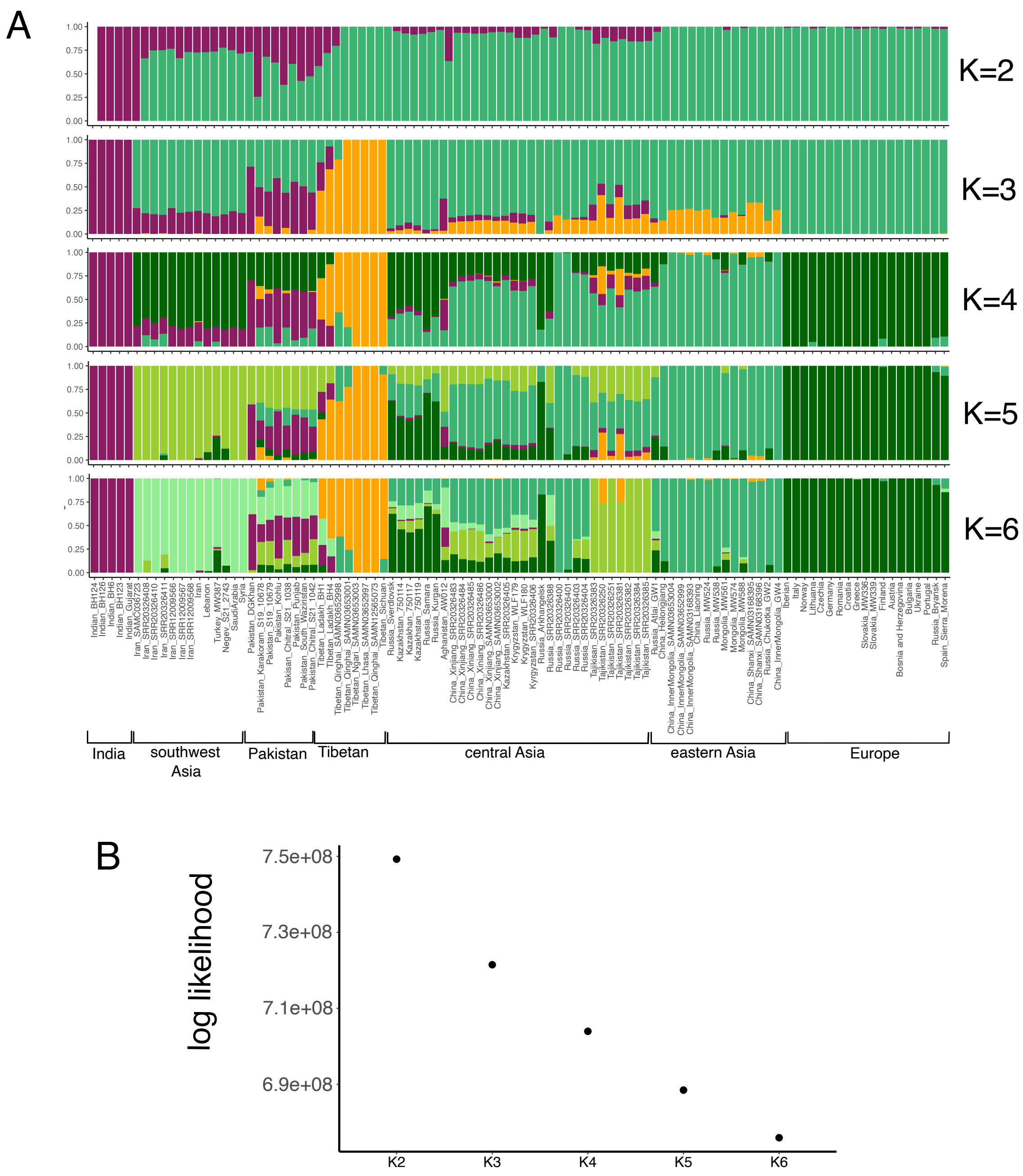

**Figure S3.** Individual admixture proportions using K=2 to K=6 populations for 98 gray wolves from Eurasia. We used 10,626,992 SNPs inferred using genotype likelihoods with ANGSD, which were used to estimate the individual admixture proportions through NGSAdmix (Skotte et al. 2013, Korneliussen et al. 2014). **(A)** Each bar represents an individual and colors within each bar represent the estimated ancestry belonging to a specific ancestry. **(B)** Log likelihoods of each run in NGSAdmix.

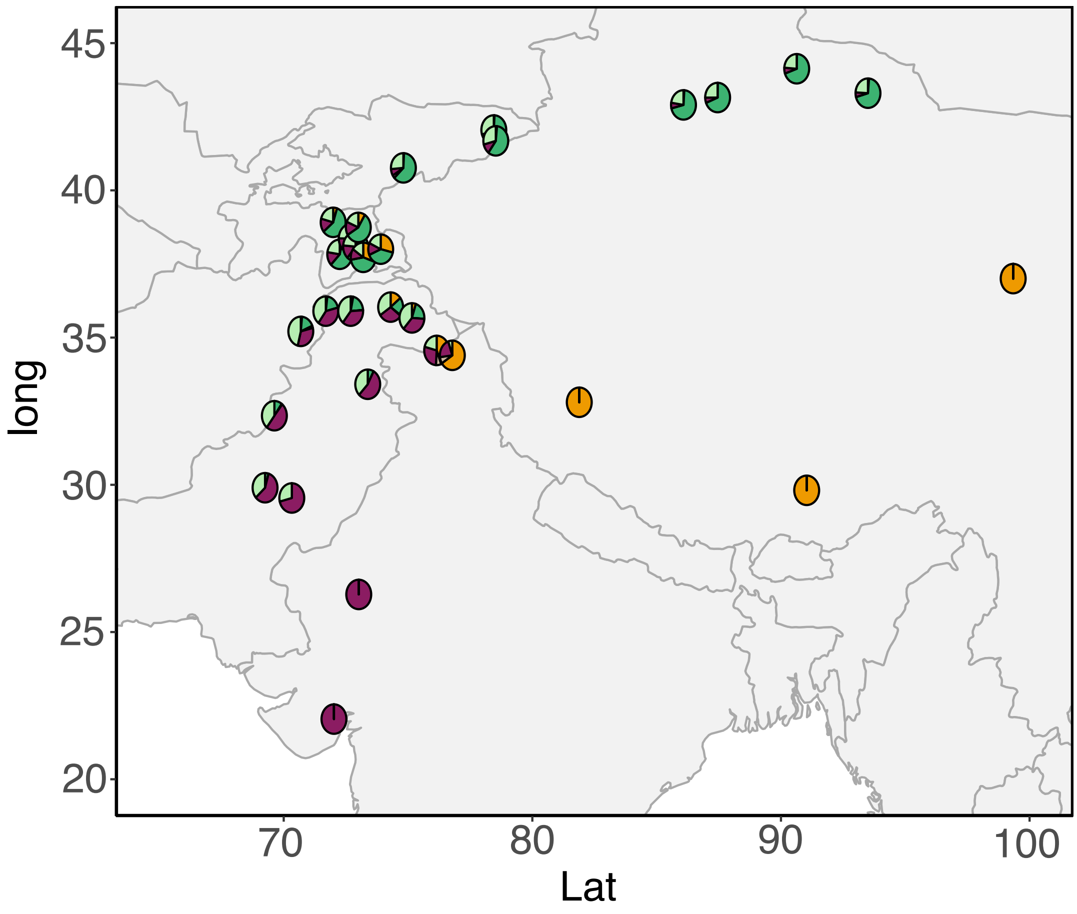


**Figure S4**. Wolf sample location on a close-up of Central and South Asia with each sample's individual admixture proportions at K=6 for 98 wolves across Eurasia. The

admixture proportions were estimated using 101 gray wolves, 4 dogs, and 9 individuals from five other canid species.

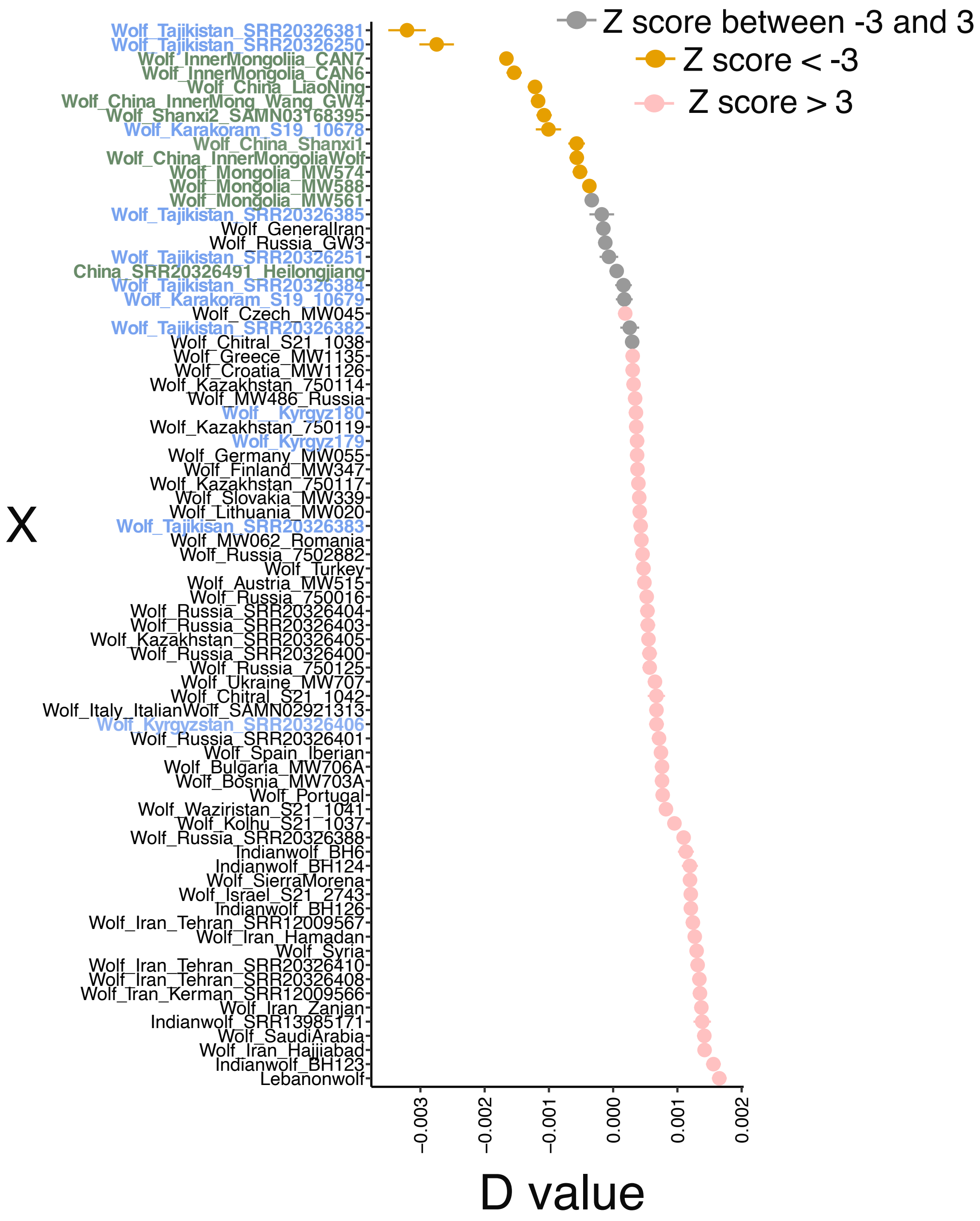


**Figure S5.** D statistic values to assess derived allele sharing between each wolf individual in Eurasia (X) with the Tibetan wolf with the topology: (Graywolf$_{Norway}$, X), Tibetan wolf–TI32), Andeanfox). A negative D-value indicates an excess derived allele sharing with the Tibetan wolf or between the Norwegian wolf and the outgroup, Andean fox. A positive D value can indicate allele sharing between X and the Andean fox, or X and the Gray wolf from Norway. Yellow and pink color indicates D values that show statistically significant derived allele sharing (Z-score < -3 or > -3). Green and blue labeled wolf samples indicate wolves that from the eastern Asia and central Asian mountains, respectively.

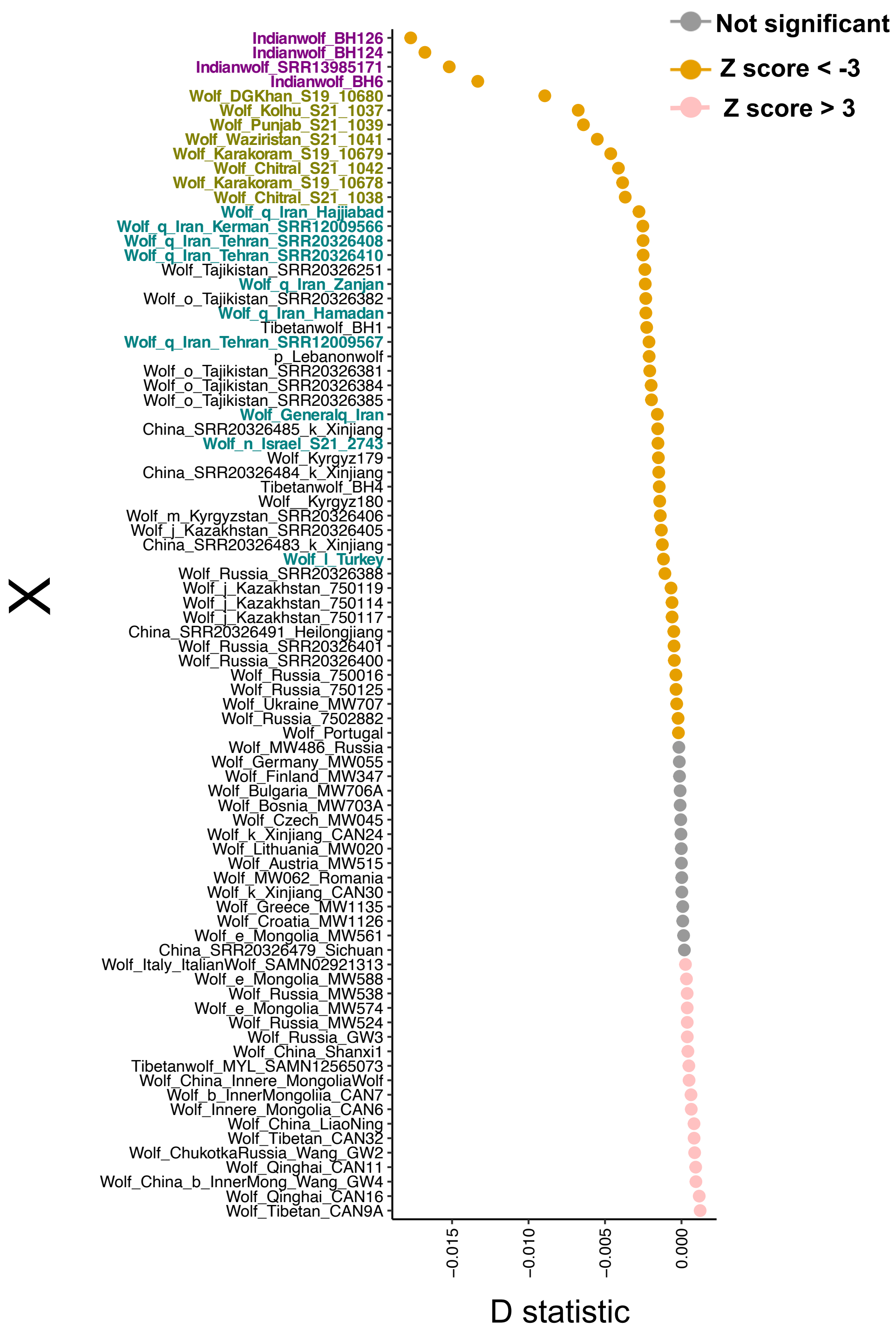


**Figure S6.** D statistic values to assess derived allele sharing between each wolf individual X with the Indian wolf with the topology: (Graywolf$_{Norway}$, X), Indian wolf BH123), Andeanfox). A negative D-value indicates an excess derived allele sharing with the Indian wolf and/or between the Norwegian wolf and the outgroup, Andean fox. A positive D value can indicate allele sharing between X and the Andean fox, or X and the gray wolf from Norway. Yellow and pink color indicates D values that show statistically significant derived allele sharing (Z-score < -3 or > -3).

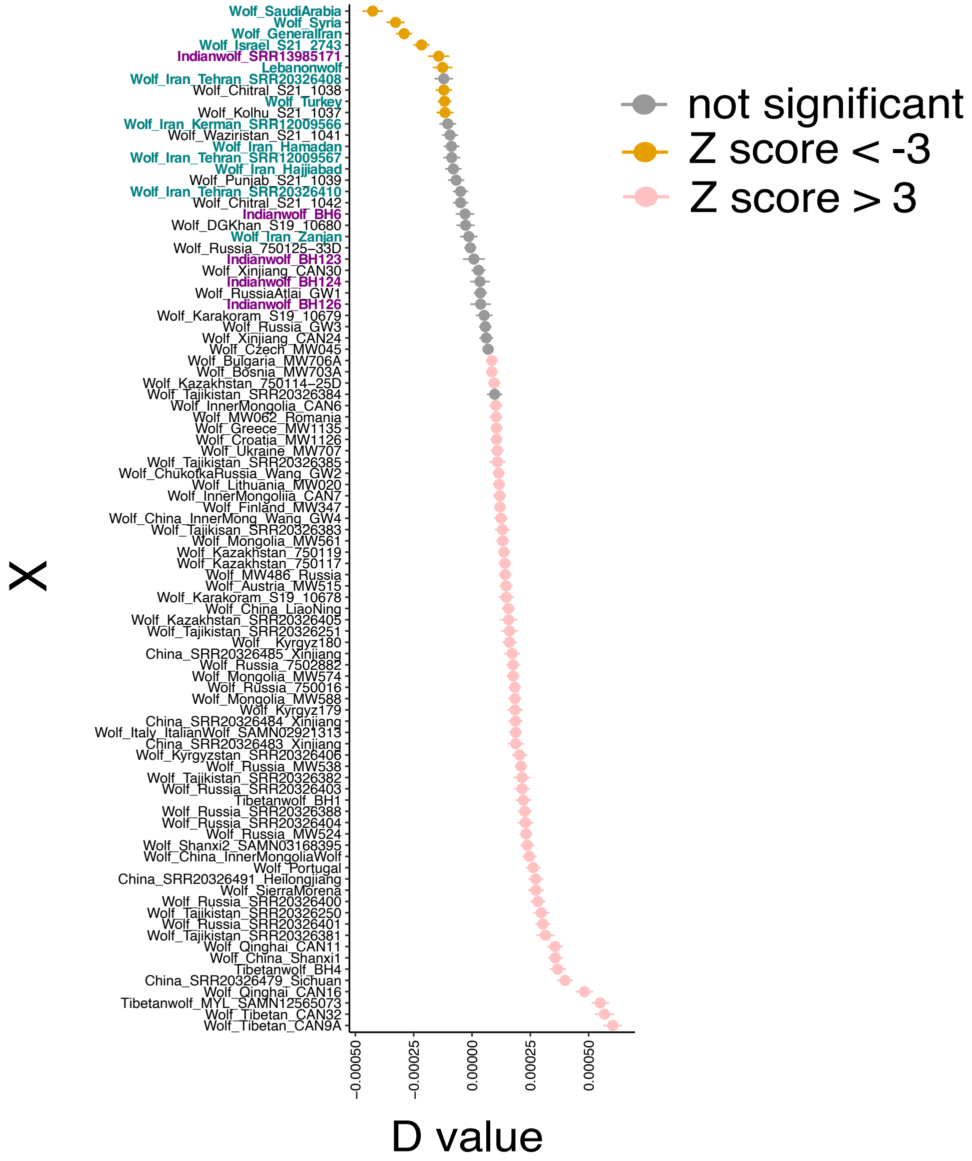


**Figure S7.** D statistic values to assess derived allele sharing between each wolf individual (X) with African wolves with the topology: (Graywolf$_{Norway}$, X), African wolf), Andeanfox). We used 4 African wolf genomes from Kenya, Morocco, Algeria, and Ethiopia as a population for P3. A negative D-value indicates an excess derived allele sharing with African wolves and/or between the Norwegian wolf and the outgroup, Andean fox. A positive D value can indicate allele sharing between X and the Andean fox, or X and the Gray wolf from Norway. Yellow and pink color indicates D values that show statistically significant derived allele sharing (Z-score < -3 or > -3). Wolves in purple indicate those that belong to the Indian lineage, greenish blue to Southwest Asia, and yellow are those that are from Pakistan.

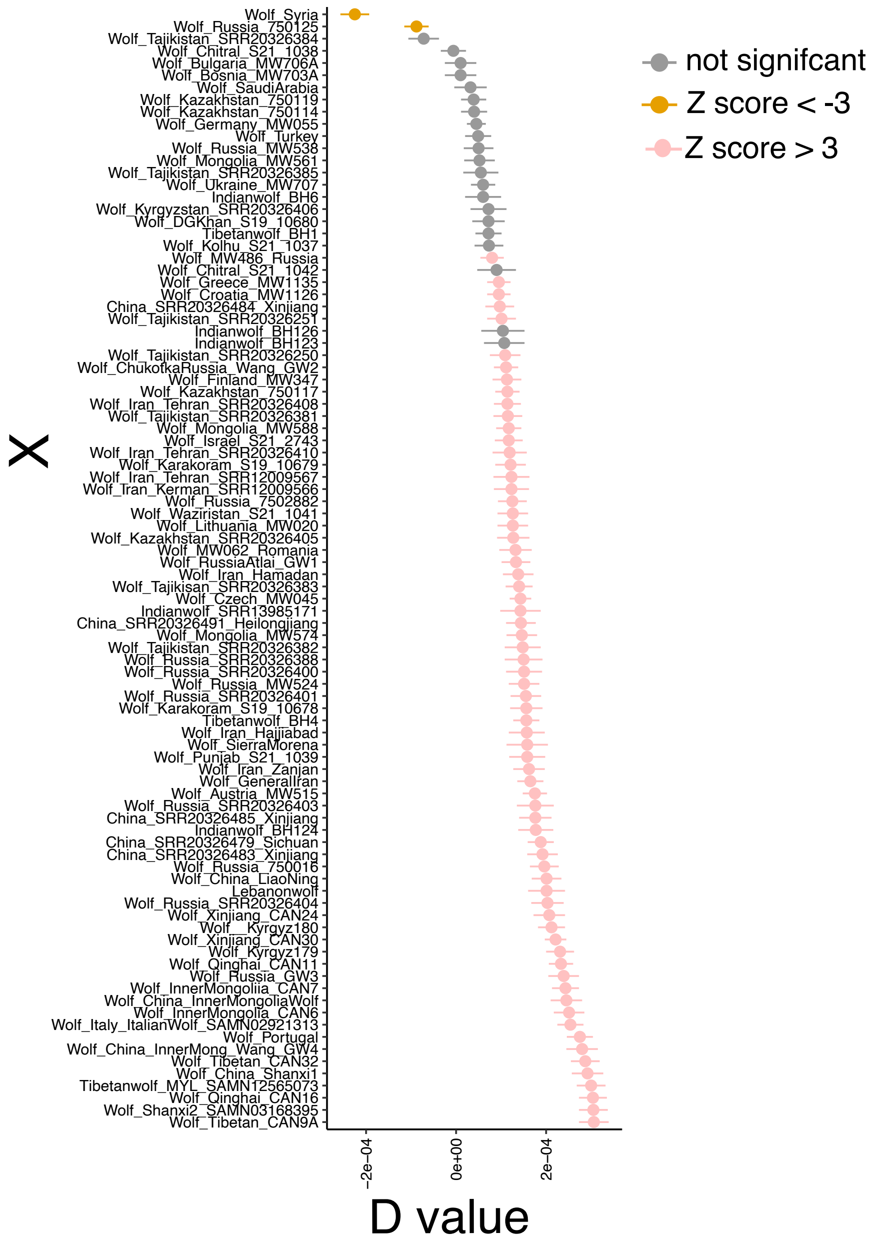


**Figure S8.** D statistic values to assess derived allele sharing between each wolf individual (X) with golden jackals with the topology: ($Graywolf_{Norway}$, X), Golden jackal), Andeanfox). We used the Golden jackal from Syria for P3. A negative D-value indicates an excess derived allele sharing with the Golden jackal and/or between the Norwegian wolf and the outgroup, Andean fox. A positive D value can indicate allele sharing between X and the Andean fox, or X and the Gray wolf from Norway. Yellow and pink color indicates D values that show statistically significant derived allele sharing (Z-score < -3 or > -3).

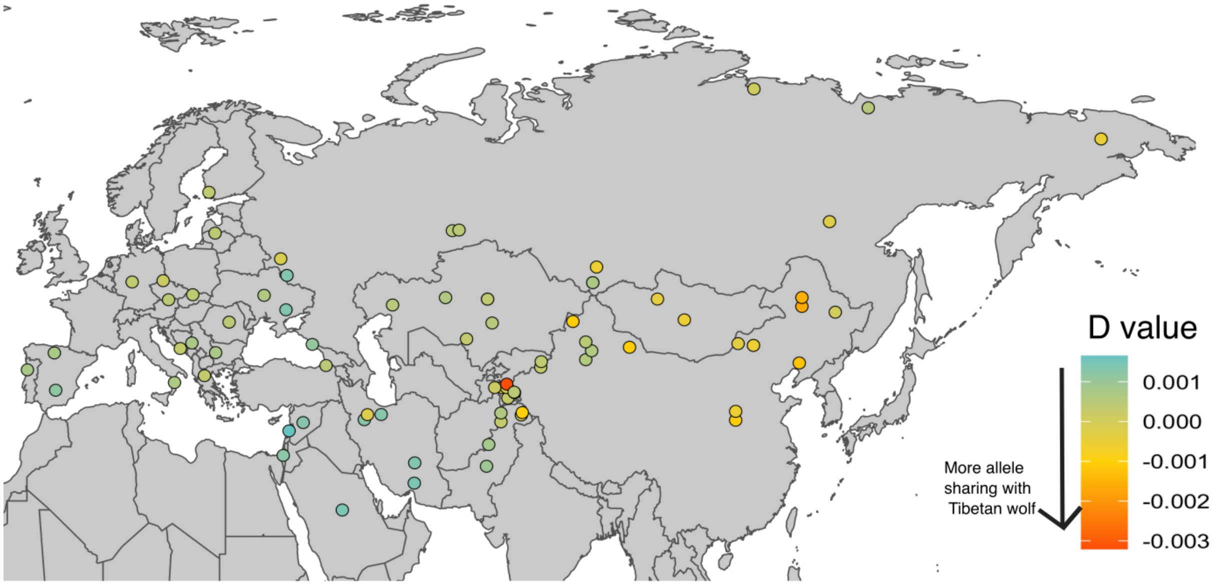


**Figure S9.** Derived allele sharing between each wolf individual in Eurasia (circles) with the Tibetan wolf with the topology: (Graywolf$_{Norway}$, X), Tibetan wolf–TI32), Andeanfox). A negative D-value indicates an excess derived allele sharing with the Tibetan wolf, while a positive D value can indicate allele sharing between X and the Andean fox, or X and the Gray wolf from Norway.

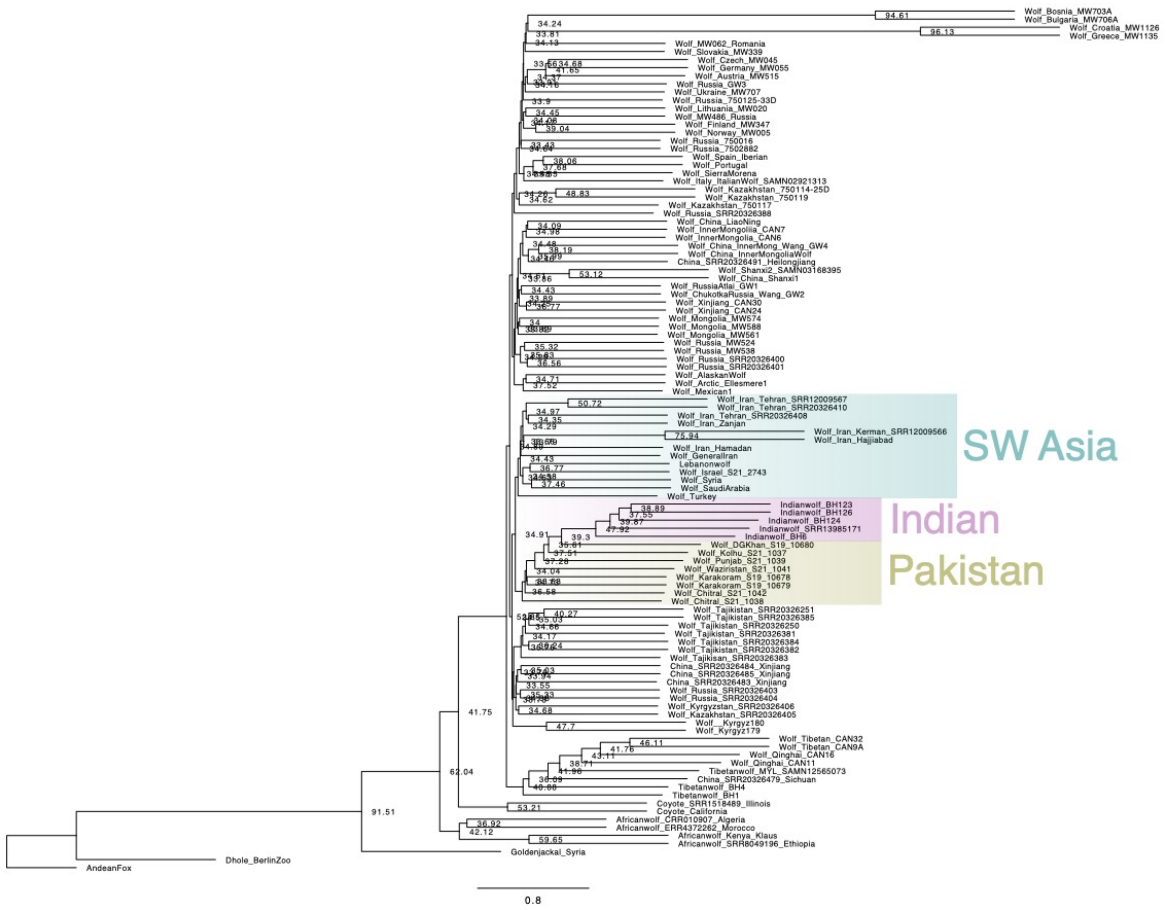


**Figure S10.** Fully labeled autosomal phylogeny inferred a multispecies coalescent tree in ASTRAL using 1,000 randomly-selected regions with a length of 20kb across the autosomes using 107 individuals. Normalized quartet score of selected nodes are shown.

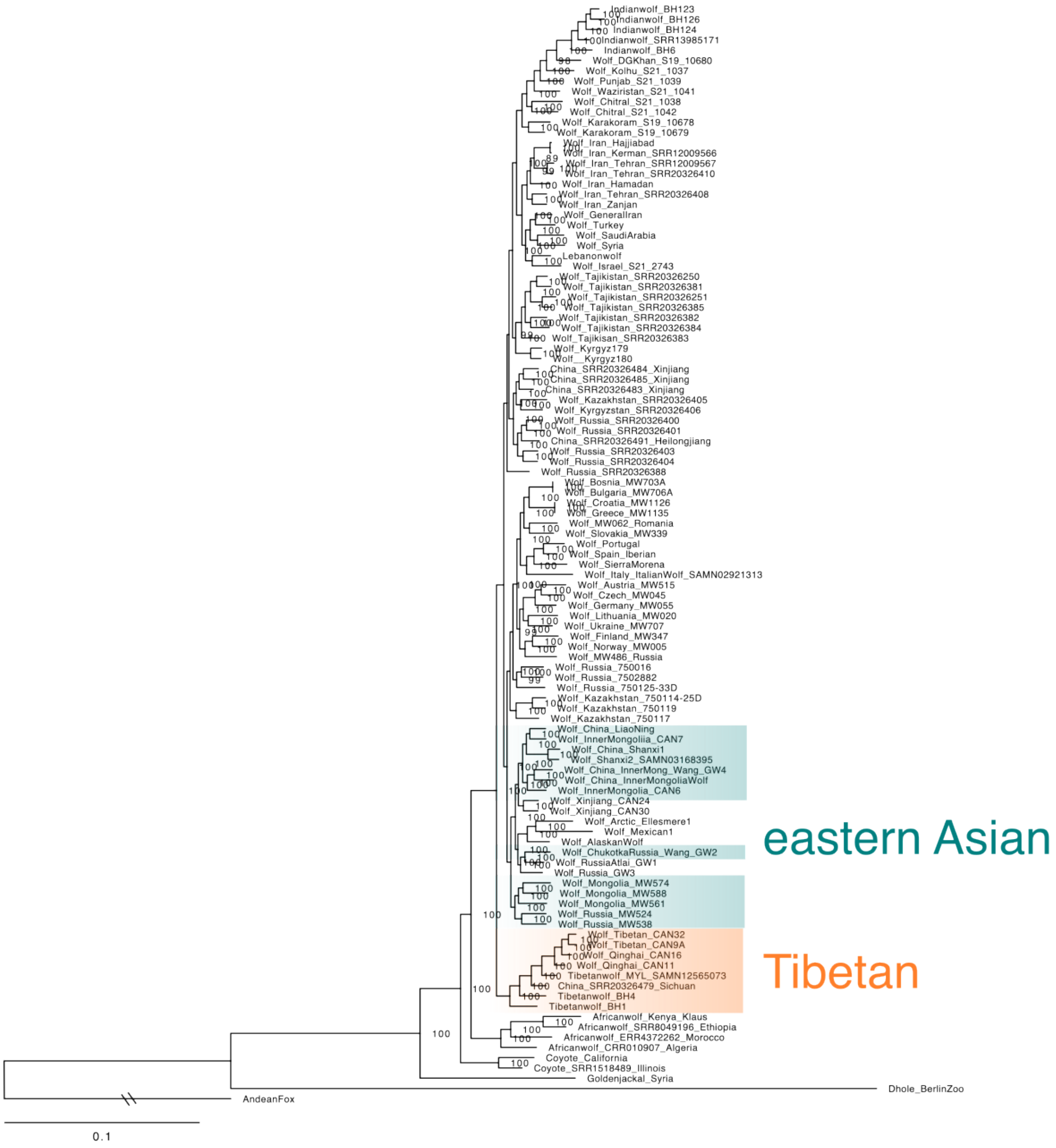


**Figure S11**. Fully labeled phylogeny using only low recombination regions (<0.2cM/Mb) across the autosomes, consisting of 3,625,481 SNPs. We ran IQ-Tree 1.6.12 with estimating the best model using ModelFinder, which was GTR+F+R5, and used 1,000 ultra-fast bootstraps to infer each tree (Kalyaanamoorthy et al. 2017, Nguyen et al. 2014). The ultra-fast bootstrap support values are shown, where a 95 value of support corresponds to a probability of 95% the clade is correct.

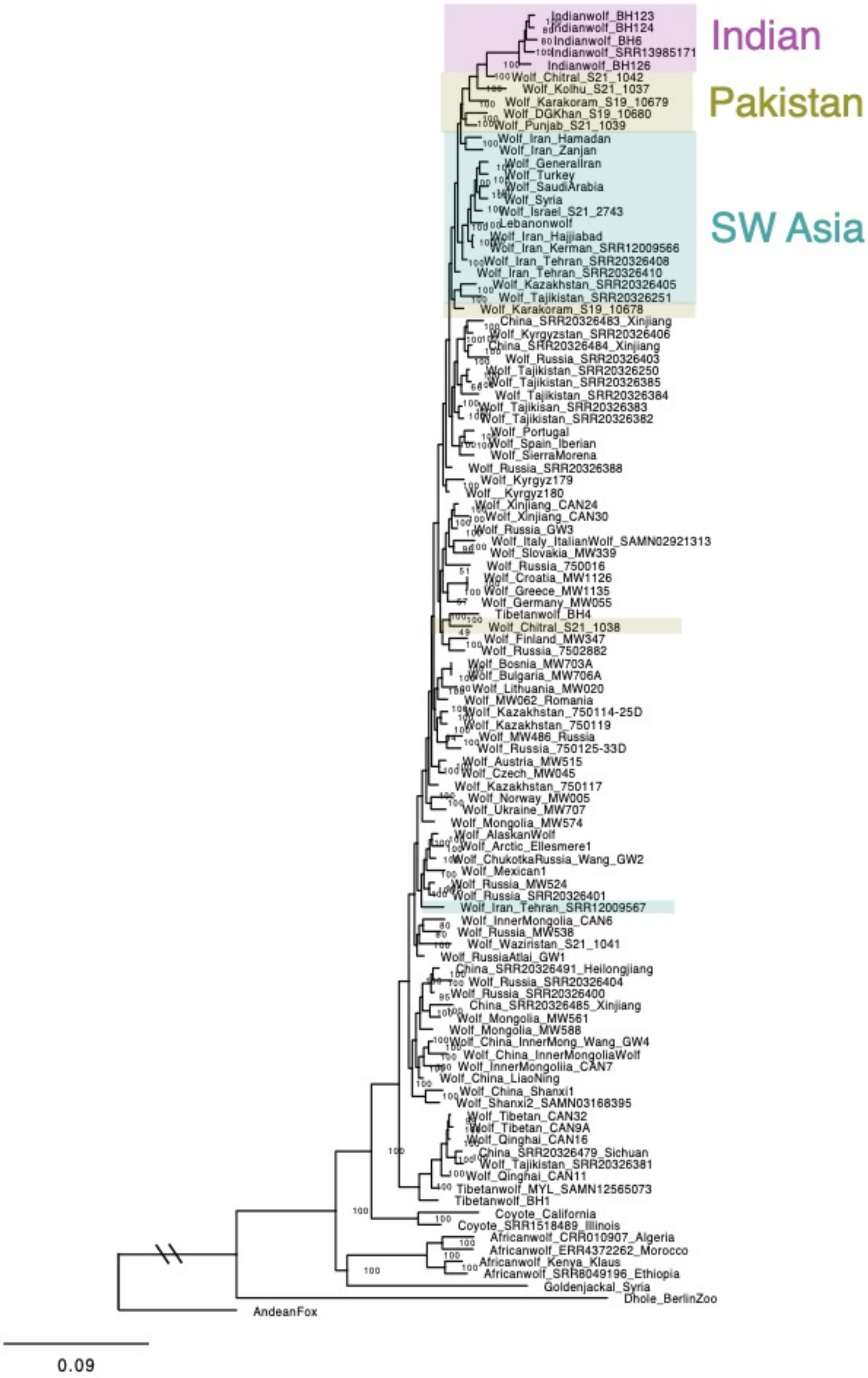

Indian
Pakistan
SW Asia
Indianwolf_BH123
Indianwolf_BH124
Indianwolf_BH6
Indianwolf_SRR13985171
Indianwolf_BH126
Wolf_Chitral_S21_1042
Wolf_Kolhu_S21_1037
Wolf_Karakoram_S19_10679
Wolf_DGKhan_S19_10680
Wolf_Punjab_S21_1039
Wolf_Iran_Hamadan
Wolf_Iran_Zanjan
Wolf_GeneralIran
Wolf_Turkey
Wolf_SaudiArabia
Wolf_Syria
Wolf_Israel_S21_2743
Lebanonwolf
Wolf_Iran_Hajjiabad
Wolf_Iran_Kerman_SRR12009566
Wolf_Iran_Tehran_SRR20326408
Wolf_Iran_Tehran_SRR20326410
Wolf_Kazakhstan_SRR20326405
Wolf_Tajikistan_SRR20326251
Wolf_Karakoram_S19_10678
China_SRR20326483_Xinjiang
Wolf_Kyrgyzstan_SRR20326406
China_SRR20326484_Xinjiang
Wolf_Russia_SRR20326403
Wolf_Tajikistan_SRR20326250
Wolf_Tajikistan_SRR20326385
Wolf_Tajikistan_SRR20326384
Wolf_Tajikisan_SRR20326383
Wolf_Tajikistan_SRR20326382
Wolf_Portugal
Wolf_Spain_Iberian
Wolf_SierraMorena
Wolf_Russia_SRR20326388
Wolf_Kyrgyz179
Wolf__Kyrgyz180
Wolf_Xinjiang_CAN24
Wolf_Xinjiang_CAN30
Wolf_Russia_GW3
Wolf_Italy_ItalianWolf_SAMN02921313
Wolf_Slovakia_MW339
Wolf_Russia_750016
Wolf_Croatia_MW1126
Wolf_Greece_MW1135
Wolf_Germany_MW055
Tibetanwolf_BH4
Wolf_Chitral_S21_1038
Wolf_Finland_MW347
Wolf_Russia_7502882
Wolf_Bosnia_MW703A
Wolf_Bulgaria_MW706A
Wolf_Lithuania_MW020
Wolf_MW062_Romania
Wolf_Kazakhstan_750114-25D
Wolf_Kazakhstan_750119
Wolf_MW486_Russia
Wolf_Russia_750125-33D
Wolf_Austria_MW515
Wolf_Czech_MW045
Wolf_Kazakhstan_750117
Wolf_Norway_MW005
Wolf_Ukraine_MW707
Wolf_Mongolia_MW574
Wolf_AlaskanWolf
Wolf_Arctic_Ellesmere1
Wolf_ChukotkaRussia_Wang_GW2
Wolf_Mexican1
Wolf_Russia_MW524
Wolf_Russia_SRR20326401
Wolf_Iran_Tehran_SRR12009567
Wolf_InnerMongolia_CAN6
Wolf_Russia_MW538
Wolf_Waziristan_S21_1041
Wolf_RussiaAtlai_GW1
China_SRR20326491_Heilongjiang
Wolf_Russia_SRR20326404
Wolf_Russia_SRR20326400
China_SRR20326485_Xinjiang
Wolf_Mongolia_MW561
Wolf_Mongolia_MW588
Wolf_China_InnerMong_Wang_GW4
Wolf_China_InnerMongoliaWolf
Wolf_InnerMongoliia_CAN7
Wolf_China_LiaoNing
Wolf_China_Shanxi1
Wolf_Shanxi2_SAMN03168395
Wolf_Tibetan_CAN32
Wolf_Tibetan_CAN9A
Wolf_Qinghai_CAN16
China_SRR20326479_Sichuan
Wolf_Tajikistan_SRR20326381
Wolf_Qinghai_CAN11
Tibetanwolf_MYL_SAMN12565073
Tibetanwolf_BH1
Coyote_California
Coyote_SRR1518489_Illinois
Africanwolf_CRR010907_Algeria
Africanwolf_ERR4372262_Morocco
Africanwolf_Kenya_Klaus
Africanwolf_SRR8049196_Ethiopia
Goldenjackal_Syria
Dhole_BerlinZoo
AndeanFox
0.09

**Figure S12**. Fully labeled phylogenetic tree of the X chromosome using 513,963 SNPs found in only the low recombination regions (<0.2cM/Mb) of 107 canid individuals. We ran IQ-Tree 1.6.12 with estimating the best model using ModelFinder, which was TVM+F+R4, and used 1,000 ultra-fast bootstraps to infer each tree (Kalyaanamoorthy et al. 2017, Nguyen et al. 2014). The ultra-fast bootstrap support values are shown, where a 95 value of support corresponds to a probability of 95% the clade is correct.

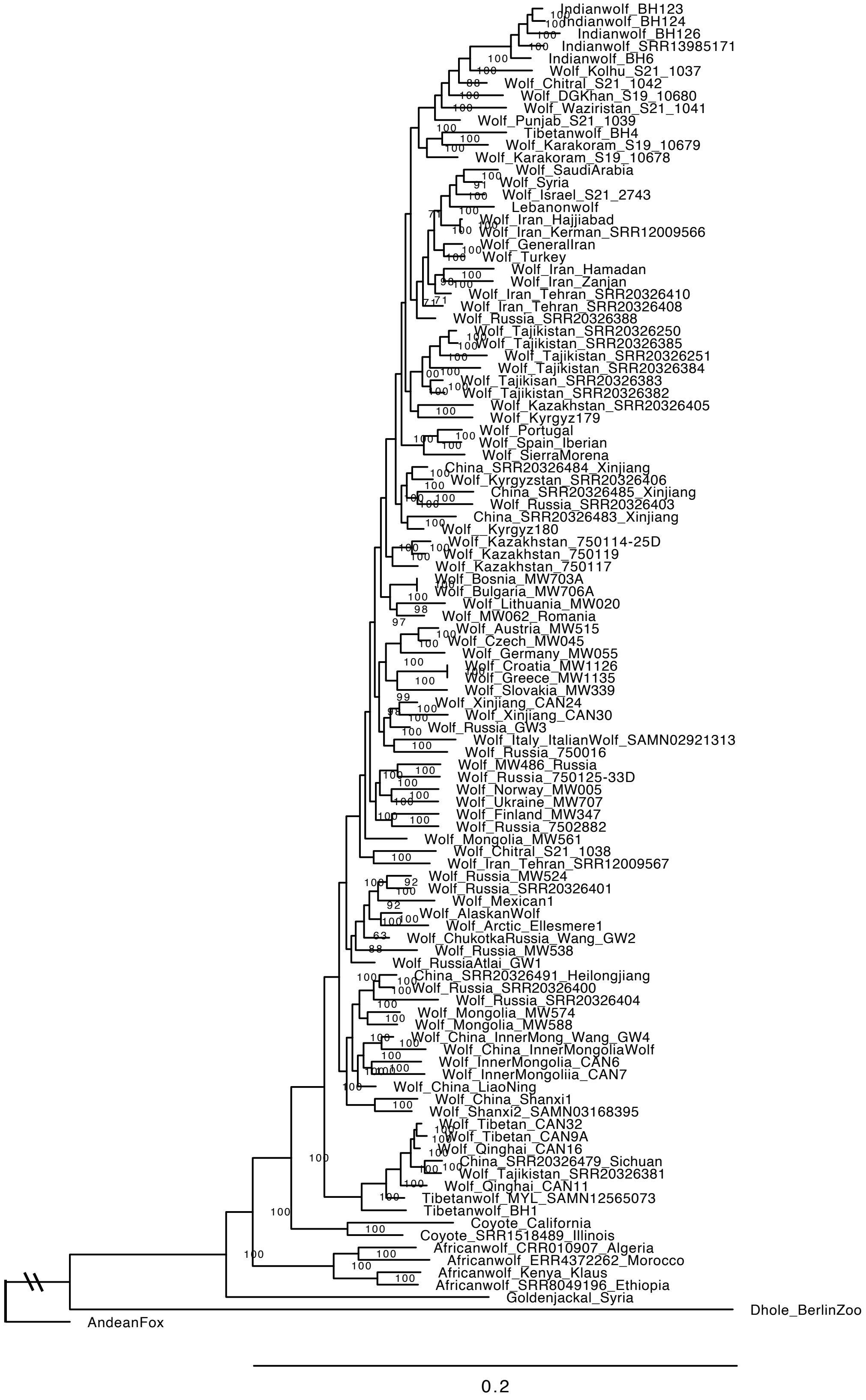
Indianwolf_BH123
Indianwolf_BH124
Indianwolf_BH126
Indianwolf_SRR13985171
Indianwolf_BH6
Wolf_Kolhu_S21_1037
Wolf_Chitral_S21_1042
Wolf_DGKhan_S19_10680
Wolf_Waziristan_S21_1041
Wolf_Punjab_S21_1039
Tibetanwolf_BH4
Wolf_Karakoram_S19_10679
Wolf_Karakoram_S19_10678
Wolf_SaudiArabia
Wolf_Syria
Wolf_Israel_S21_2743
Lebanonwolf
Wolf_Iran_Hajjiabad
Wolf_Iran_Kerman_SRR12009566
Wolf_GeneralIran
Wolf_Turkey
Wolf_Iran_Hamadan
Wolf_Iran_Zanjan
Wolf_Iran_Tehran_SRR20326410
Wolf_Iran_Tehran_SRR20326408
Wolf_Russia_SRR20326388
Wolf_Tajikistan_SRR20326250
Wolf_Tajikistan_SRR20326385
Wolf_Tajikistan_SRR20326251
Wolf_Tajikistan_SRR20326384
Wolf_Tajikisan_SRR20326383
Wolf_Tajikistan_SRR20326382
Wolf_Kazakhstan_SRR20326405
Wolf_Kyrgyz179
Wolf_Portugal
Wolf_Spain_Iberian
Wolf_SierraMorena
China_SRR20326484_Xinjiang
Wolf_Kyrgyzstan_SRR20326406
China_SRR20326485_Xinjiang
Wolf_Russia_SRR20326403
China_SRR20326483_Xinjiang
Wolf__Kyrgyz180
Wolf_Kazakhstan_750114-25D
Wolf_Kazakhstan_750119
Wolf_Kazakhstan_750117
Wolf_Bosnia_MW703A
Wolf_Bulgaria_MW706A
Wolf_Lithuania_MW020
Wolf_MW062_Romania
Wolf_Austria_MW515
Wolf_Czech_MW045
Wolf_Germany_MW055
Wolf_Croatia_MW1126
Wolf_Greece_MW1135
Wolf_Slovakia_MW339
Wolf_Xinjiang_CAN24
Wolf_Xinjiang_CAN30
Wolf_Russia_GW3
Wolf_Italy_ItalianWolf_SAMN02921313
Wolf_Russia_750016
Wolf_MW486_Russia
Wolf_Russia_750125-33D
Wolf_Norway_MW005
Wolf_Ukraine_MW707
Wolf_Finland_MW347
Wolf_Russia_7502882
Wolf_Mongolia_MW561
Wolf_Chitral_S21_1038
Wolf_Iran_Tehran_SRR12009567
Wolf_Russia_MW524
Wolf_Russia_SRR20326401
Wolf_Mexican1
Wolf_AlaskanWolf
Wolf_Arctic_Ellesmere1
Wolf_ChukotkaRussia_Wang_GW2
Wolf_Russia_MW538
Wolf_RussiaAtlai_GW1
China_SRR20326491_Heilongjiang
Wolf_Russia_SRR20326400
Wolf_Russia_SRR20326404
Wolf_Mongolia_MW574
Wolf_Mongolia_MW588
Wolf_China_InnerMong_Wang_GW4
Wolf_China_InnerMongoliaWolf
Wolf_InnerMongolia_CAN6
Wolf_InnerMongoliia_CAN7
Wolf_China_LiaoNing
Wolf_China_Shanxi1
Wolf_Shanxi2_SAMN03168395
Wolf_Tibetan_CAN32
Wolf_Tibetan_CAN9A
Wolf_Qinghai_CAN16
China_SRR20326479_Sichuan
Wolf_Tajikistan_SRR20326381
Wolf_Qinghai_CAN11
Tibetanwolf_MYL_SAMN12565073
Tibetanwolf_BH1
Coyote_California
Coyote_SRR1518489_Illinois
Africanwolf_CRR010907_Algeria
Africanwolf_ERR4372262_Morocco
Africanwolf_Kenya_Klaus
Africanwolf_SRR8049196_Ethiopia
Goldenjackal_Syria
Dhole_BerlinZoo
AndeanFox
0.2

**Figure S13**. Fully labeled phylogenetic tree of the X chromosome using 2,761,668 SNPs of 107 canid individuals. We ran IQ-Tree 1.6.12 with estimating the best model using ModelFinder, which was TVM+F+R4, and used 1,000 ultra-fast bootstraps to infer each tree (Kalyaanamoorthy et al. 2017, Nguyen et al. 2014). The ultra-fast bootstrap support values are shown, where a 95 value of support corresponds to a probability of 95% the clade is correct.

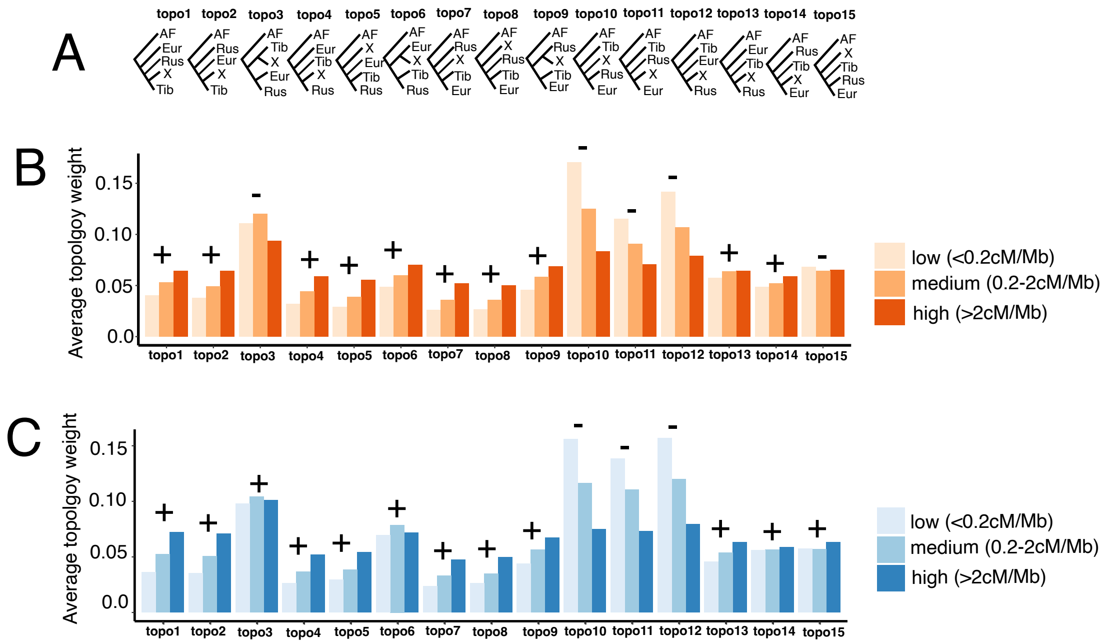


**Figure S14. (A)** Fifteen possible topologies using the Andean fox (AF) as an outgroup and four different gray wolf populations: Tibetan (Tb), Central Russia (Rus), European (Eur), and X, which denotes either east Asian wolves or high altitude Central Asian wolves. **(B)** Using east Asian wolves as X, the average topology weight for each possible topology across the X chromosome averaged within three categories: low recombination regions (<0.2cM/Mb), medium (0.2-2cM/Mb), and high (>2cM/Mb). **(C)** Using high altitude Central Asian wolves as X, the average topology weight for each possible topology across the X chromosome averaged within three categories: low recombination regions (<0.2cM/Mb), medium (0.2-2cM/Mb), and high (>2cM/Mb). Signs refer to whether the average within the high recombination region is larger (+) or smaller (-) than the average in the low recombination region.

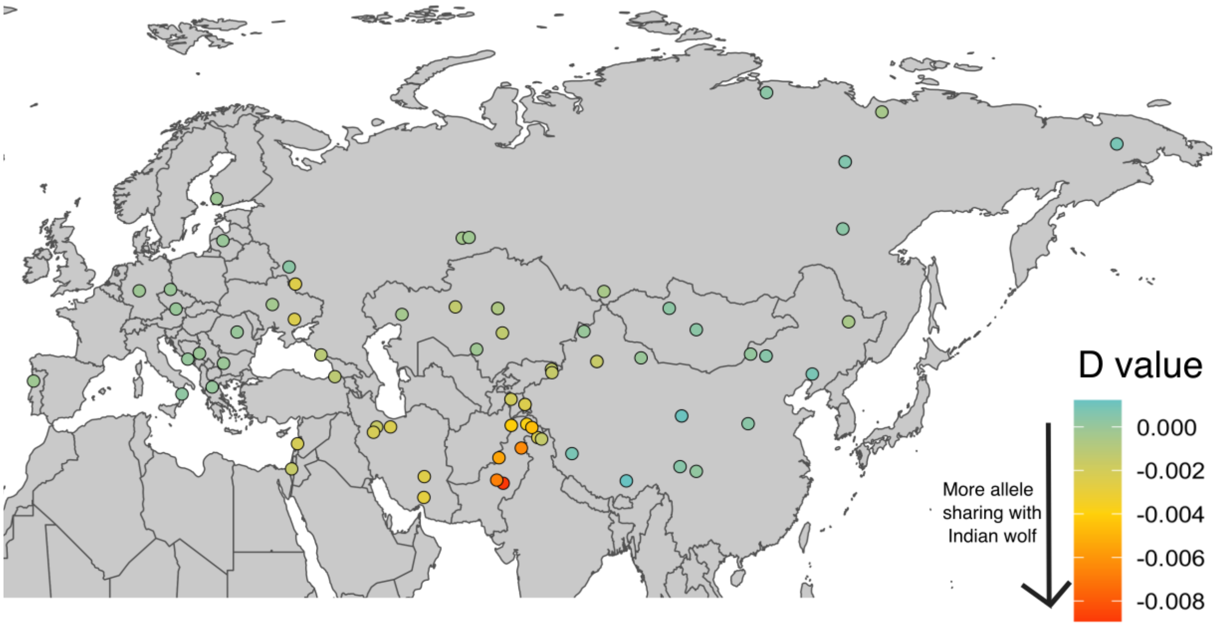


**Figure S15.** Derived allele sharing between each wolf individual in Eurasia (circles) with the Indian wolf with the topology: (Graywolf$_{Norway}$, X), Indian wolf–BH123), Andeanfox). A negative D-value indicates an excess derived allele sharing with the Indian wolf and/or between X the Gray wolf from Norway and the Andean fox, while a positive D value can indicate allele sharing between X and the Andean fox, or X and the Gray wolf from Norway.

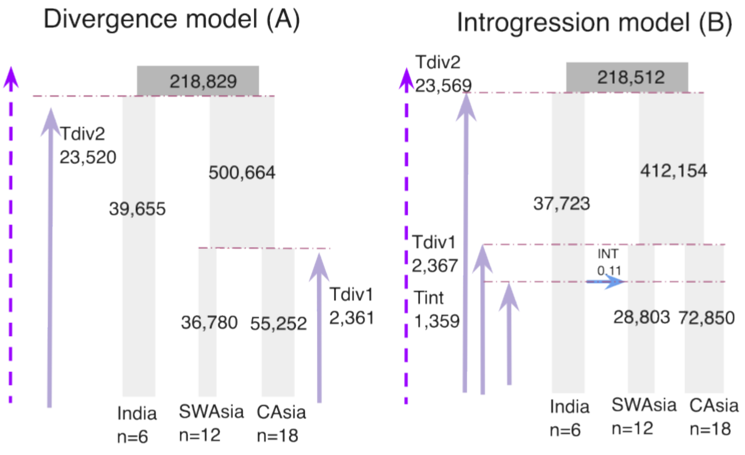


**Figure S16.** Three alternative scenarios for the origin of the Southwest Asian population tested in fastsimcoal2 (Excoffier et al. 2021). The width of the gray bars and the numbers within the bars represent the population size of each population (A) A simple bifurcation model in which southwest Asia and central Asia diverged simultaneously from an ancestral Asian population; (B) an introgression model in which the present southwest Asian population was formed by introgression from India and central Asian wolves; (C) an hybridization model in which southwest Asia is an hybrid formed through admixture between wolves from India and central Asia. Note that model B is nested in model A, reducing A when INT=0.

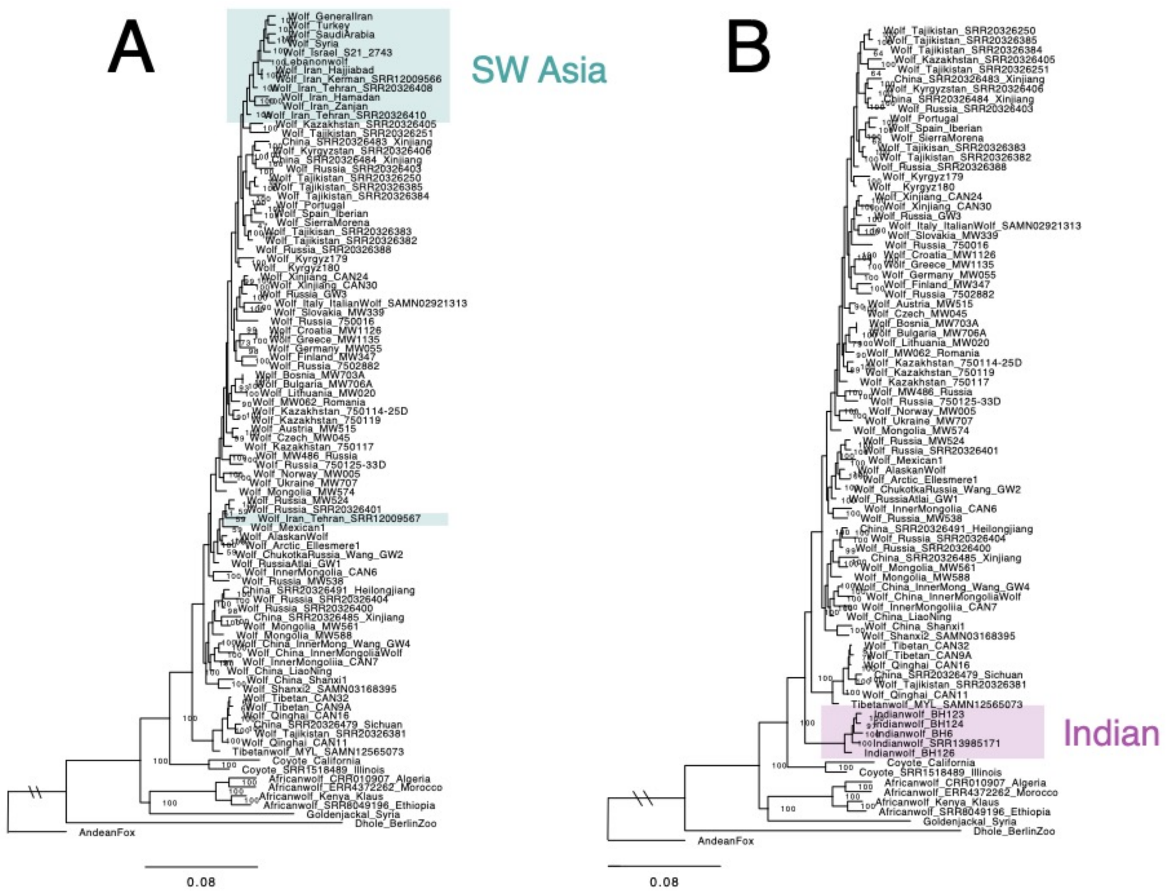


**Figure S17**. (A, B) Maximum likelihood phylogeny of wild canids inferred with IQ-Tree 1.6.12 using only low recombination (<0.2cM/Mb) regions of the X chromosome. We used IQ-Tree 1.6.12 where we estimated the best model using ModelFinder and used 1,000 ultra-fast bootstraps to infer each tree. The phylogenetic relationships vary depending on which wolf individuals are included: (A) has no wolves from Pakistan and India, and (B) has no wolves from Pakistan and southwestern Asia. For tree A and B, the best model was TVM+F+R3.

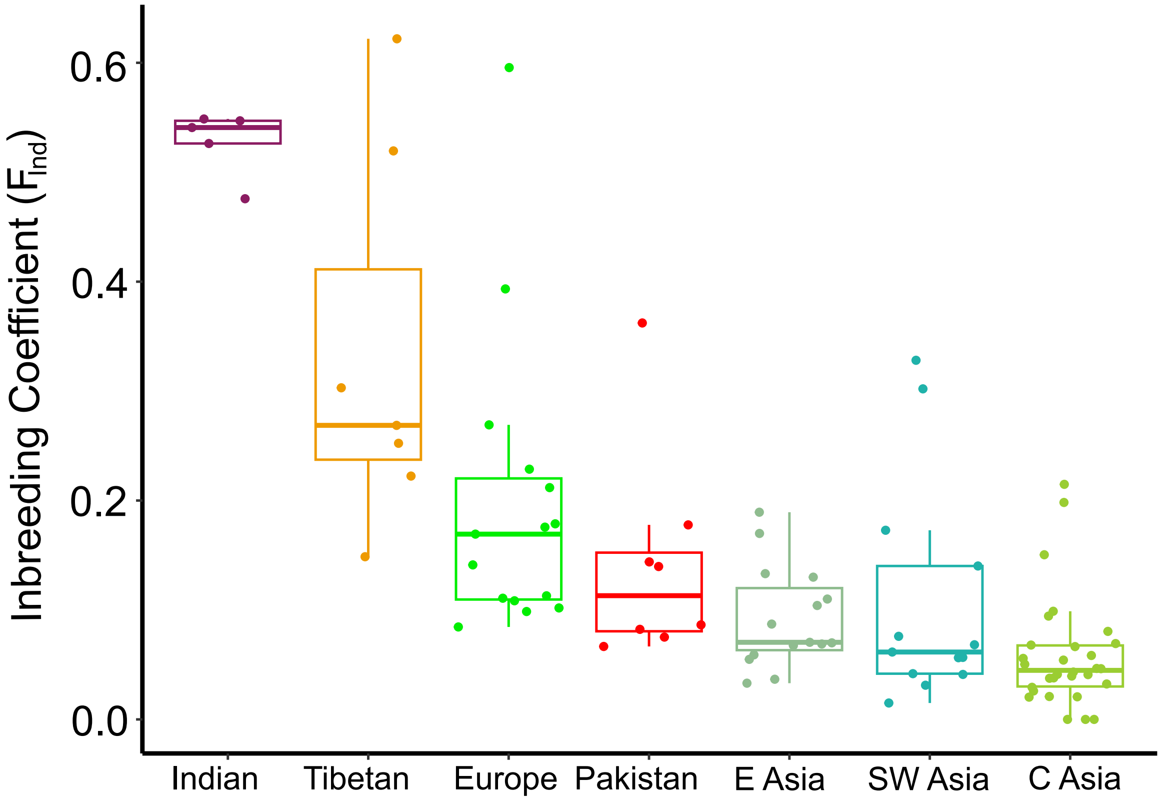


**Figure S18**. Estimated inbreeding coefficients ($F_{Ind}$) using genotype likelihoods with NgsRelate for 96 individuals across seven wolf populations in Eurasia.

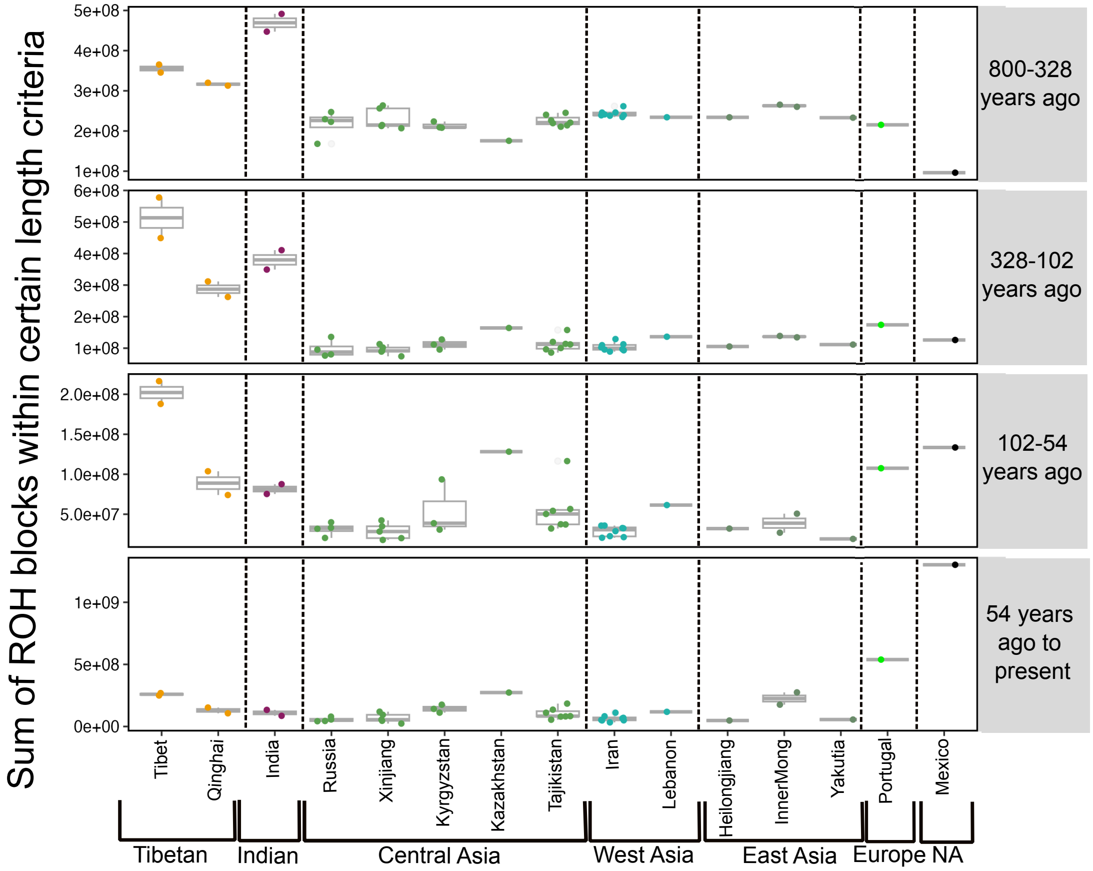


**Figure S19.** Timing of inbreeding estimated from the length of ROH blocks across the autosomes of 40 wolves. Inbreeding timings between 820-328 years ago corresponding to ROH lengths of 200Kb-500Kb, 328-102 years ago corresponding to ROH lengths of 500Kb-1.6Mb, 102-54 years ago corresponding to ROH lengths of 1.6Mb-3Mb, and 54 years ago to present corresponding to ROH lengths above 3Mb. Wolves most representing the Indian and Himalayan lineages generally show historical inbreeding in the last 800 years compared to wolves from Mexico and Portugal, which show recent inbreeding in the 100 years.

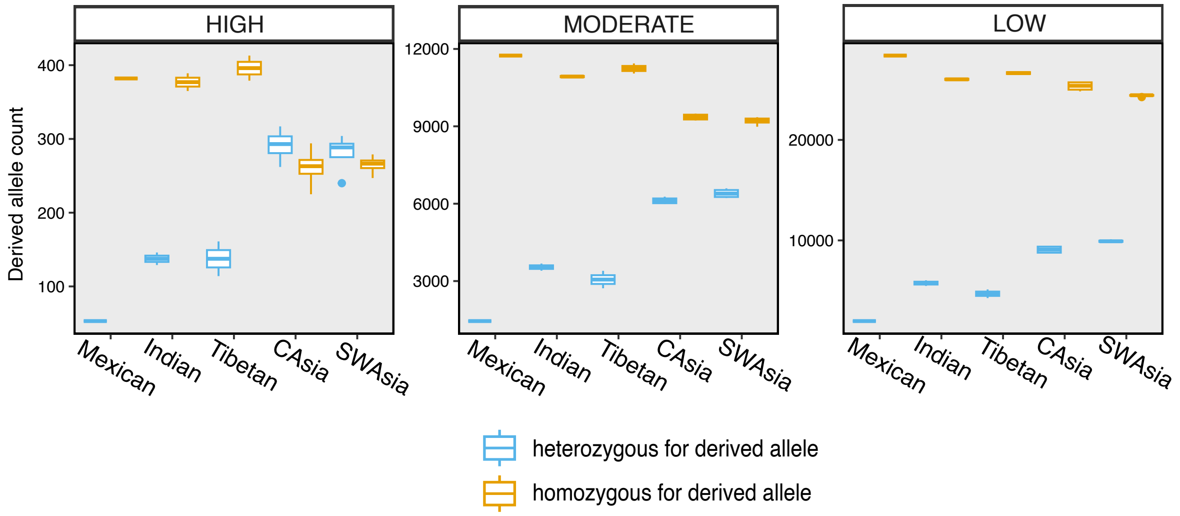


**Figure S20.** Number of heterozygous and homozygous counts for the derived allele for High impact, Medium impact, and Low impact categories. Homozygous counts contain two derived alleles and heterozygous derived counts contain one derived allele. For High and Moderate categories, we find Mexican, Indian, and Tibetan wolves have higher counts of homozygous derived alleles, consistent with genome-wide measures of higher homozygosity.

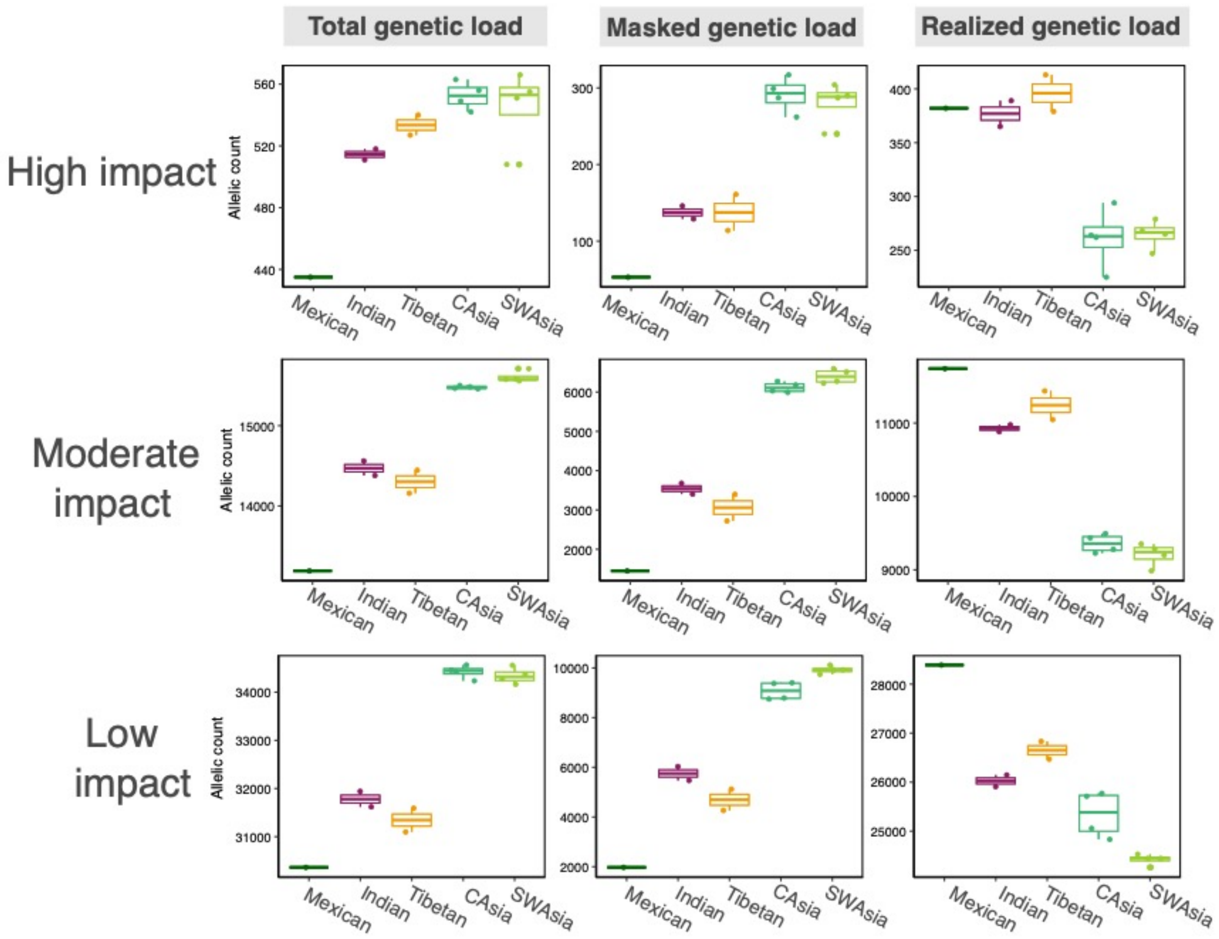


**Figure S21.** Total genetic load (the total number of derived alleles; homozygous derived alleles counted twice, heterozygous derived alleles once), realized load (homozygous state of derived alleles), and masked load (heterozygous state of derived alleles) for each impact category (High, Moderate, Low) for five selected wolf populations of wolves. For all categories, we find Mexican, Indian, and Tibetan wolves have a lower total genetic load than wolves in southwest Asia and central Asia. Because low impact is considered to be mostly harmless, the lower total genetic load in historically small wolf populations suggests it could be due to loss of total derived variants from genetic drift.

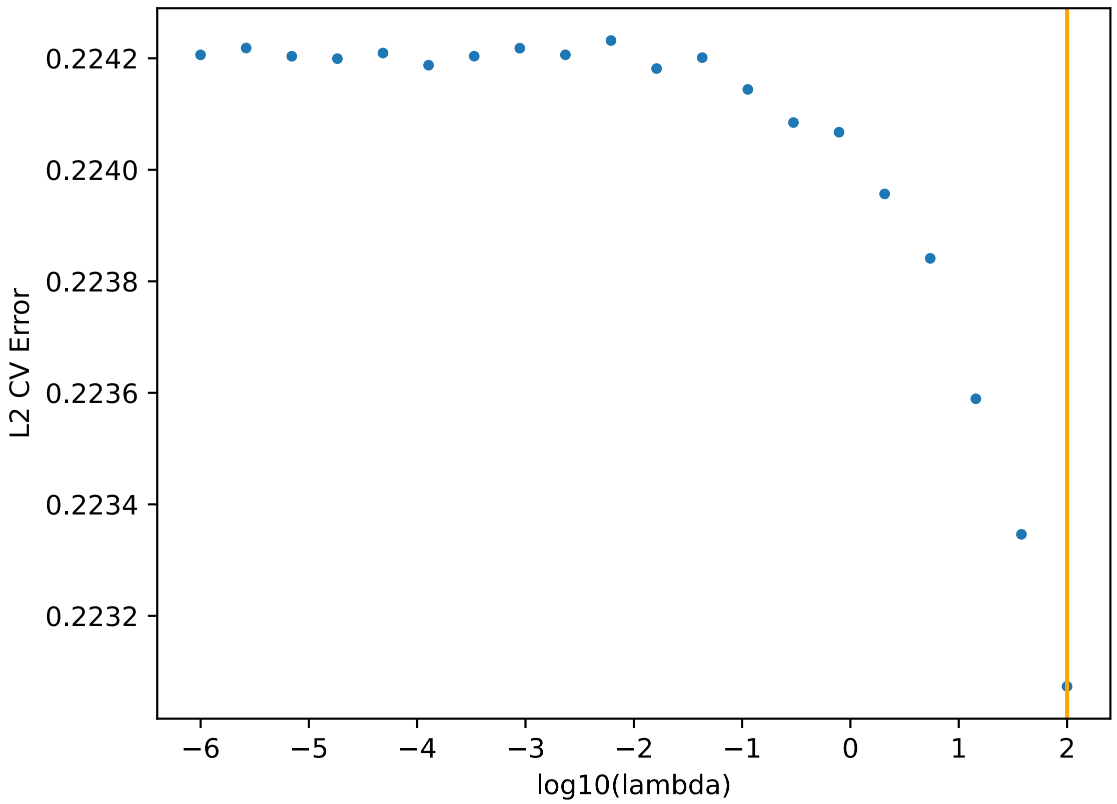


**Figure S22**. Cross validation results by using leave-one-out cross validation to select the optimal value of lambda, the smoothing parameter. A lambda of 100 was selected to be the optimal value and used in the final analysis.

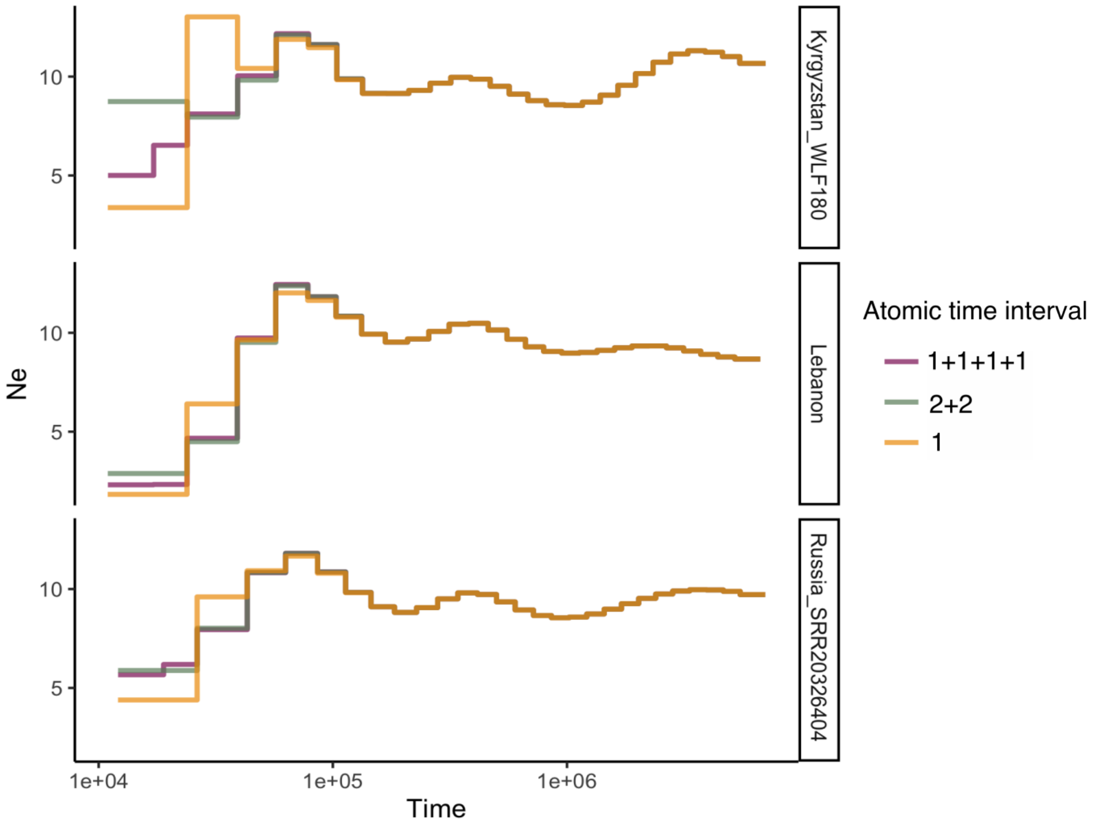


**Figure S23.** PSMC plots for three wolf genomes while varying the atomic time intervals. We observe erroneous peaks for the Kyrgyzstan WLF180 genome when using atomic time interval 1, whereas this peak disappears when using 2+2 or 1+1+1+1. In our study, we use the atomic time interval of 1+1+1+1 to avoid false PSMC peaks.

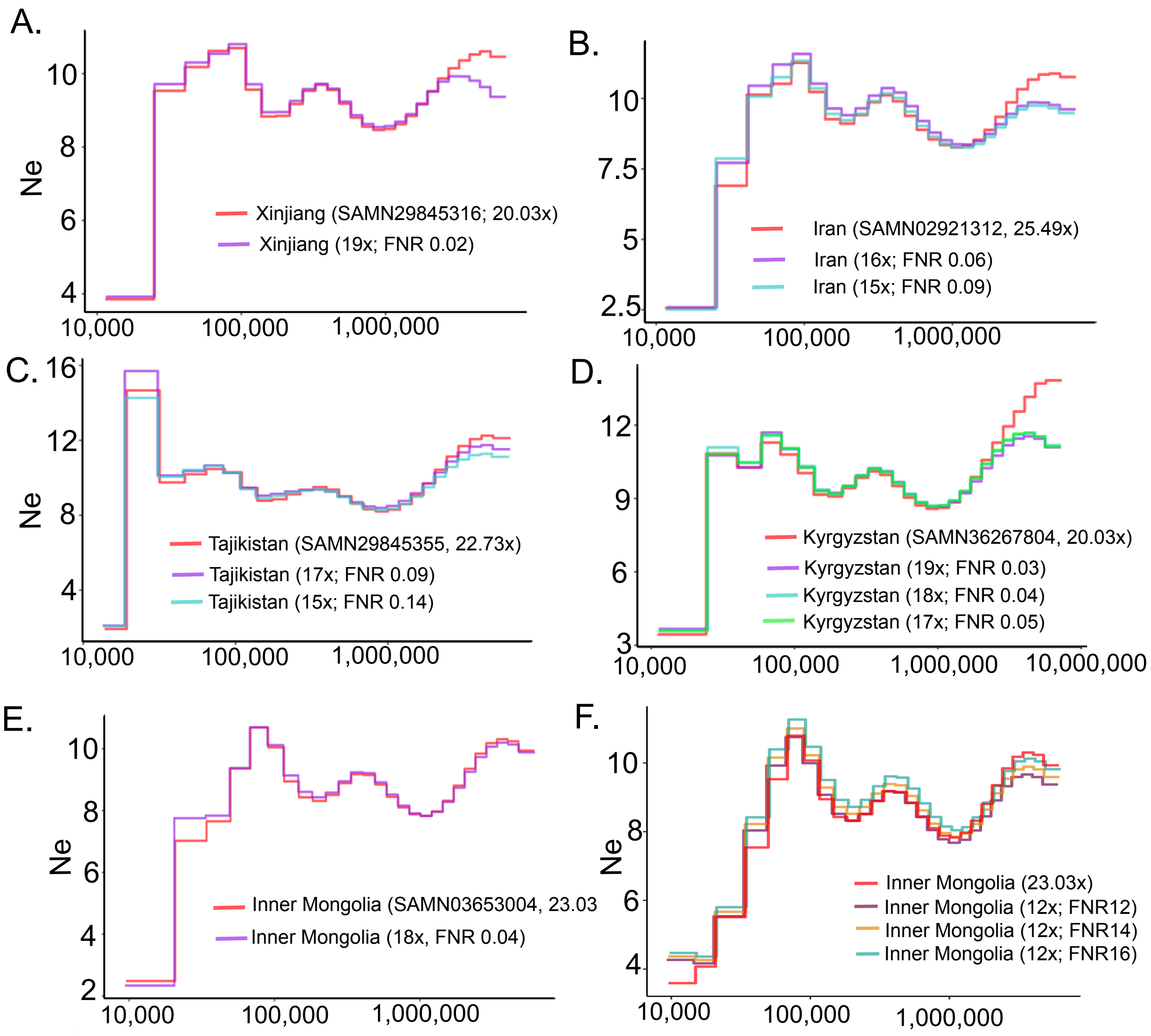


**Figure S24**. Down-sampling of genomes to select the false negative rates (FNR) for low-depth corrections of pairwise sequentially Markovian coalescent (PSMC) demographic trajectories. High coverage wolf genomes (>20x) that were from the same region as the target low coverage samples were selected, which consisted of genomes from Xinjiang (SAMN29845316; 20.03x), Iran (SAMN02921312, 25.49x), Inner Mongolia (SAMN03653004, 23.03x), Kyrgyzstan (SAMN36267804, 25.86), and Tajikistan (SAMN29845355, 22.73x). Panel F was used to select the false negative rate (FNR) for the wolf genome from Shanxi, China, which was at a coverage of 12x.

**Figure S25.** Est and Tpl input files for fastsimcoal2 for each of the three models we tested.

**Model 1: 3PopDiv**
**Description**: 3PopDiv ('Strictly biforcating'): One single population diverged into two pops (India and Asia) at a given time TSPLIT in the past, and after that, at time TDIV1, there was a split of the 'Asia' population into two populations (CAsia and SWAsia) so that at present time there is three populations: India SWAsia and CAsia

**3PopDiv.est**

---

```
// Search ranges and rules file
// ****************************

[PARAMETERS]
//#isInt? #name   #dist.#min  #max
//all Ns are in number of haploid individuals
1  $NCAS$      unif  2e3   4e5   output
1  $NIND$      unif  2e3   4e5   output
1  $NSWAS$      unif  2e3   4e5   output
1  $NASANC$      unif 2e3   4e5   output
1  $NANC$      unif  2e3   4e5   output

1  $TSPLIT$   unif  20000    50000  output bounded
1  $TDIV1$      unif  2325   $TSPLIT$     output bounded paramInRange

[COMPLEX PARAMETERS]
```

---

**3PopDiv.tpl**

---

```
//Parameters for the coalescence simulation program : fsimcoal2.exe
3 samples to simulate :
//Population effective sizes (number of genes)
$NCAS$ //cAsia
$NIND$                                  //India
$NSWAS$                                 //swAsia
//Samples sizes and samples age
18
6
12
//Growth rates                          : negative growth implies population expansion
0
0
0
//Number of migration matrices : 0 implies no migration between demes
0
//historical event: time, source, sink, migrants, new deme size, new growth rate, migration matrix index
```

2 historical event
$TDIV1$ 0 2 1 $NASANC$ 0 0 absoluteResize
$TSPLIT$ 2 1 1 $NANC$ 0 0 absoluteResize
//Number of independent loci [chromosome]
1 0
//Per chromosome: Number of contiguous linkage Block: a block is a set of contiguous loci
1
//per Block:data type, number of loci, per generation recombination and mutation rates and optional parameters
FREQ 1 0 4.5e-9 OUTEXP

---

**<u>Model 2: </u>3PopIntrog**

**Description**: 3PopIntrog ('Introgression Model'): One single population diverged into two pops (India and Asia) at a given time TSPLIT in the past, and after that, at time TDIV1, there was a split of the 'Asia' population into two populations (CAsia and SWAsia) so that at present time there is India SWAsia and CAsia. At a given time TINTROG in the past there was introgression from India to SWAsia (proportion INT)

### 3PopIntrog.est

---

// Search ranges and rules file
// ****************************

[PARAMETERS]
//#isInt? #name #dist.#min #max
//all Ns are in number of haploid individuals
1 $NCAS$ unif 2e3 4e5 output
1 $NIND$ unif 2e3 4e5 output
1 $NSWAS$ unif 2e3 4e5 output
1 $NASANC$ unif 2e3 4e5 output
1 $NANC$ unif 2e3 4e5 output

1 $TSPLIT$ unif 20000 50000 output bounded
1 $TDIV1$ unif 2325 $TSPLIT$ output bounded paramInRange
1 $TINTROG$ unif 1 $TDIV$ output bounded paramInRange

0 $INT$ logunif 0.000000001 1 output bounded

[COMPLEX PARAMETERS]

---

### 3PopIntrog.tpl

---

//Parameters for the coalescence simulation program : fsimcoal2.exe
3 samples to simulate :
//Population effective sizes (number of genes)
$NCAS$ //cAsia

$NIND$ //India
$NSWAS$ //swAsia
//Samples sizes and samples age
18
6
12
//Growth rates : negative growth implies population expansion
0
0
0
//Number of migration matrices : 0 implies no migration between demes
0
//historical event: time, source, sink, migrants, new deme size, new growth rate, migration matrix index
3 historical event
$TINTROG$ 2 1 $INT$ 1 0 0
$TDIV$ 0 2 1 $NASANC$ 0 0 absoluteResize
$TSPLIT$ 2 1 1 $NANC$ 0 0 absoluteResize
//Number of independent loci [chromosome]
1 0
//Per chromosome: Number of contiguous linkage Block: a block is a set of contiguous loci
1
//per Block:data type, number of loci, per generation recombination and mutation rates and optional parameters
FREQ 1 0 4.5e-9 OUTEXP

---

**Model 3: 3PopHyb**

**Description:** 3PopHyb ('Hybrid origin'): One single pop diverged into two pops India and CAsia at a given time TSPLIT in the past, and after that at time TINTROG a new hybrid population (SWasia) was formed by the contribution of INT from India and (1-INT) from CAsia.

**3PopHyb.est**

---

// Search ranges and rules file
// ****************************

[PARAMETERS]
//#isInt? #name #dist.#min #max
//all Ns are in number of haploid individuals
1 $NCAS$ unif 2e3 4e5 output
1 $NIND$ unif 2e3 4e5 output
1 $NSWAS$ unif 2e3 4e5 output
1 $NANC$ unif 2e3 4e5 output

1 $TSPLIT$ unif 20000 50000 output bounded
1 $TINTROG$ unif 1 $TSPLIT$ output bounded paramInRange

0 $INT$ logunif 0.000000001 1 output bounded

[COMPLEX PARAMETERS]
1 $TINTROG20$ = $TINTROG$+1 hide

---

**3PopHyb.tpl**

---

//Parameters for the coalescence simulation program : fsimcoal2.exe
3 samples to simulate :
//Population effective sizes (number of genes)
$NCAS$ //cAsia
$NIND$ //India
$NSWAS$ //swAsia
//Samples sizes and samples age
18
6
12
//Growth rates : negative growth implies population expansion
0
0
0
//Number of migration matrices : 0 implies no migration between demes
0
//historical event: time, source, sink, migrants, new deme size, new growth rate, migration matrix index
3 historical event
$TINTROG$ 2 1 $INT$ 1 0 0
$TINTROG20$ 2 0 1 1 0 0
$TSPLIT$ 1 0 1 $NANC$ 0 0 absoluteResize
//Number of independent loci [chromosome]
1 0
//Per chromosome: Number of contiguous linkage Block: a block is a set of contiguous loci
1
//per Block:data type, number of loci, per generation recombination and mutation rates and optional parameters
FREQ 1 0 4.5e-9 OUTEXP

---

**Supplementary Table 1.** Estimated likelihoods for the three demographic models. The likelihood corresponds to the maximum likelihood computed under each model in 100 simulations. 'Log10(L) 1SNP' corresponds to the likelihood computed from the 3D-SFS obtained from 1SNP per block and without the monomorphic sites. In contrast, log10(L) corresponds to the likelihood obtained when considering all SNPs.

| Model | **$Log_{10}$(L)** | **$Log_{10}$(L) 1SNP** | **#Param** | **AIC** | **relat L** |
|---|---|---|---|---|---|
| A.Bifurcation | -12686767.32 | -970.29 | 7 | 4482.37 | 0.34 |
| **B.Introgression** | **-12671308.13** | **-969.15** | **9** | **4481.12** | **0.64** |
| C.Hybridization | -12675514.76 | -971.5 | 7 | 4487.93 | 0.02 |

**Supplementary Table 2**. Demographic parameters and their search ranges as used in the FASTSIMCOAL2 analyses. Note that certain parameters are constrained by an upper limit preventing them from exceeding this threshold (bounded). In particular, the divergence time (under the bifurcation model) and hybridization time (under the hybridization model) are parameters whose upper limits are constrained by the estimated split time. Also, introgression time (TINT) is upper bounded at the Southwestern Asia and Central Asia divergence time (TDIV1) in model B and bounded at TDIV2 in model C. Other parameters are permitted to surpass the maximum value defined within the search range. All parameters, except for hybridization and introgression proportions, are sampled from uniform distributions. The introgression proportions are sampled from log-uniform distributions. Time is in units of generations and all population sizes are in the number of haploid individuals (2N).

| Parameter | Distribution | Minimum | Maximum | Bounded |
|---|---|---|---|---|
| NPOP0 | Uniform | $2 \times 10^3$ | $4 \times 10^5$ | no |
| NIND | Uniform | $2 \times 10^3$ | $4 \times 10^5$ | no |
| NSWAS | Uniform | $2 \times 10^3$ | $4 \times 10^5$ | no |
| NCAS | Uniform | $2 \times 10^3$ | $4 \times 10^5$ | no |
| NANC | Uniform | $2 \times 10^3$ | $4 \times 10^5$ | no |
| NASANC | Uniform | $2 \times 10^3$ | $4 \times 10^5$ | no |
| TDIV1 | Uniform | $2 \times 10^3$ | TSPLIT | yes |
| TDIV2 | Uniform | $2 \times 10^4$ | $5 \times 10^4$ | yes |
| TINTROG | Uniform | 10 | TDIV | yes |
| INT | Log-uniform | $1 \times 10^{-9}$ | 1 | yes |

## Supplementary References


Ersmark E, Klutsch CFC, Chan YL, Sinding MHS, Fain SR, Illarionova NA, Oskarsson M, Uhlen M, Zhang UP, Dalen L, Savolainen P. 2016. From the past to the present: wolf phylogeography and demographic history based on the Mitochondrial control region. Frontiers in Ecology and Evolution 10.3389.

Hoffmann, M. & Atickem, A. 2019. *Canis lupaster*. *The IUCN Red List of Threatened Species* 2019: e.T118264888A118265889

Werhahn G, Liu Y, Meng Y, Cheng C, Lu Z, Atzeni L, Deng Z, Kun S, Shao X, Joshi J, Sherchan M, Karmacharya D, et al. 2020. Himalayan wolf distribution and admixture based on multiple genetic markers. Journal of Biogeography 47(6): 1272-1285.

Werhahn, G., Hennelly, L.M., Lyngdoh, S.J., Habib, B., Viranta, S. & Shrotriya, S. 2024. *Canis lupus* ssp. *chanco*. *The IUCN Red List of Threatened Species* 2024: e.T223987824A258477123.

Hennelly LM, Sarwar G, Fatima H, Werhahn G, Abbas FI, Khan AM, Mahmood T, Kachel S, Kubanychbekov Z, Waseem MT, et al. 2023. Genomic analysis of wolves from Pakistan clarifies boundaries among three divergent wolf lineages. 115:339-348.

Wang GD, Zhang M, Wang X, Yang MA, Cao P, Liu F, Lu H, Feng X, Skoglund P, Wang L, Fu Q, Zhang YP. 2019. Genomic approaches reveal an endemic subpopulation of gray wolves in Southern China. iScience 20:110-118.

Wang L, Ma YP, Zhou QJ, Zhang YP, Savolainen P, Wang GD. 2016. The geographical distribution of grey wolves (Canis lupus) in China: a systematic review. Zoological Research 37(6): 315-326.
