# Supplementary material for: Continent-wide view of genomic diversity and divergence in the wolves of Asia
Source: Commun Biol. 2025 Dec 24;9:330. doi: 10.1038/s42003-025-09379-9 (PMC12953585; doi:10.1038/s42003-025-09379-9)
Supplement: Supplementary file 2 — Description of Additional Supplementary Materials [file 42003_2025_9379_MOESM2_ESM.pdf]

Description of Additional Supplementary Files

File name: Supplementary Data 1

Description: Sample list and associated metadata for newly sequenced and previously published canid genomes included in this study
