# Supplementary material for: Continent-wide view of genomic diversity and divergence in the wolves of Asia
Source: Commun Biol. 2025 Dec 24;9:330. doi: 10.1038/s42003-025-09379-9 (PMC12953585; doi:10.1038/s42003-025-09379-9)
Supplement: Supplementary file 4 — Reporting Summary [file 42003_2025_9379_MOESM4_ESM.pdf]

nature portfolio

Corresponding author(s): Lauren Hennelly

Last updated by author(s): Nov 14, 2025

Reporting Summary

Nature Portfolio wishes to improve the reproducibility of the work that we publish. This form provides structure for consistency and transparency in reporting. For further information on Nature Portfolio policies, see our Editorial Policies and the Editorial Policy Checklist.

Statistics

For all statistical analyses, confirm that the following items are present in the figure legend, table legend, main text, or Methods section.

n/a	Confirmed	
☐	☒	The exact sample size (*n*) for each experimental group/condition, given as a discrete number and unit of measurement
☒	☐	A statement on whether measurements were taken from distinct samples or whether the same sample was measured repeatedly
☐	☒	The statistical test(s) used AND whether they are one- or two-sided<br>*Only common tests should be described solely by name; describe more complex techniques in the Methods section.*
☒	☐	A description of all covariates tested
☒	☐	A description of any assumptions or corrections, such as tests of normality and adjustment for multiple comparisons
☐	☒	A full description of the statistical parameters including central tendency (e.g. means) or other basic estimates (e.g. regression coefficient) AND variation (e.g. standard deviation) or associated estimates of uncertainty (e.g. confidence intervals)
☒	☐	For null hypothesis testing, the test statistic (e.g. *F*, *t*, *r*) with confidence intervals, effect sizes, degrees of freedom and *P* value noted<br>*Give P values as exact values whenever suitable.*
☒	☐	For Bayesian analysis, information on the choice of priors and Markov chain Monte Carlo settings
☐	☒	For hierarchical and complex designs, identification of the appropriate level for tests and full reporting of outcomes
☒	☐	Estimates of effect sizes (e.g. Cohen's *d*, Pearson's *r*), indicating how they were calculated

Our web collection on statistics for biologists contains articles on many of the points above.

Software and code

Policy information about availability of computer code

Data collection	All scripts used in the analysis are publicly available here: https://github.com/hennelly/Asia_wide_wolf_genomics. All versions of software are given in the main manuscript.
Data analysis	All scripts used in the analysis are publicly available here: https://github.com/hennelly/Asia_wide_wolf_genomics

For manuscripts utilizing custom algorithms or software that are central to the research but not yet described in published literature, software must be made available to editors and reviewers. We strongly encourage code deposition in a community repository (e.g. GitHub). See the Nature Portfolio guidelines for submitting code & software for further information.

Data

Policy information about availability of data

All manuscripts must include a data availability statement. This statement should provide the following information, where applicable:

- Accession codes, unique identifiers, or web links for publicly available datasets
- A description of any restrictions on data availability
- For clinical datasets or third party data, please ensure that the statement adheres to our policy

All raw reads have been submitted to the National Center for Biotechnology Information under Project ID PRJNA1285574 with accession numbers SAMN49769329 -SAMN49769349. The bioinformatic scripts and results of the analyses can be found on the github: https://github.com/hennelly/Asia_wide_wolf_genomics

Research involving human participants, their data, or biological material

Policy information about studies with human participants or human data. See also policy information about sex, gender (identity/presentation), and sexual orientation and race, ethnicity and racism.

Reporting on sex and gender	NA
Reporting on race, ethnicity, or other socially relevant groupings	NA
Population characteristics	NA
Recruitment	NA
Ethics oversight	NA

Note that full information on the approval of the study protocol must also be provided in the manuscript.

Field-specific reporting

Please select the one below that is the best fit for your research. If you are not sure, read the appropriate sections before making your selection.

☐ Life sciences ☐ Behavioural & social sciences ☒ Ecological, evolutionary & environmental sciences

For a reference copy of the document with all sections, see nature.com/documents/nr-reporting-summary-flat.pdf

Ecological, evolutionary & environmental sciences study design

All studies must disclose on these points even when the disclosure is negative.

Study description	Gray wolves (Canis lupus) in Asia encompass most of the species' global genetic diversity and many endangered populations. However, a clear understanding of the evolutionary history of wolves from many parts of Asia, especially southern regions, is lacking. To address these gaps, we used 98 whole genomes of wolves sampled across Eurasia to investigate phylogenetic and gene flow histories across the genome, and to characterize their genetic diversity and demographic history. Specifically we compiled a dataset with these newly sequenced genomes and 95 previously sequenced wolves from North America (n=3) and Eurasia (n=78), as well as dogs (n=4), and 10 individuals from six other canid species: dhole, Cuon alpinus (n=1); Ethiopian wolf, Canis simensis (n=1); golden jackals, Canis aureus (n=1); African wolf, Canis lupaster (n=4); coyote, Canis latrans (n=2), Andean fox; Lycalopex culpaeus (n=1). We performed various analyses described in the main manuscript to refine our understanding of the evolutionary history of gray wolves in Asia and inform their conservation.
Research sample	We generated 20 new whole genomes from the gray wolf, Canis lupus. These samples were derived from blood, tissue, or a museum specimen, in which we extracted the DNA from these specimens. We compiled an additional 95 previously published whole genomes, which we downloaded from the National Center for Biotechnology Information (NCBI) Sequence Read Archive (SRA). We used these raw fastq files to align to the domestic dog reference genome assembly (CanFam3.1) which we downloaded from NCBI SRA.
Sampling strategy	Our sampling of new whole genomes of gray wolves was targeted in locations where there were few previously published wolf genomes sequenced. We obtained samples of as many gray wolves as possible from these regions, which in some cases was only 7 or 8 from a country as gray wolves are generally rare and difficult to obtain samples from wild wolves. We made sure to obtain multiple individuals from a population for certain analyses. These included the fastsimcoal2 analysis where we had more than 5 individuals in each population and our genetic load analysis, where we had at least 2 individuals that were high coverage (>20x). We also made sure to have adequate outgroup populations to test admixture scenarios. In these cases, we obtained representative individuals of canid species that could hybridize with the gray wolf and that overlapped with the gray wolf distribution (African wolf, golden jackal). We also ensured we had adequate canid species as an outgroup on our phylogenetic trees, which we included African wild dog, Dhole, and the gray fox. A description of which individuals were used in which analyses is provided in Table S1.
Data collection	Wild wolf samples were collected from various collaborators that captured the wild wolves in the field. In addition, wolf tissue and blood samples were sequenced from samples archived at museums (Yekaterinburg Museum in Russia and Steinhardt Museum of Natural History in Israel). Cagan H. Sekercioglu and M. Cisel Kemahi Aytekin caught the wolf from Turkey for a satellite collaring project, Ladislav Paule sampled the wolf from Slovakia, the wolves from Pakistan were sampled by Hira Fatima and Ghulam Sarwar.
Timing and spatial scale	There was no start or end date of the sampling of our newly sequenced gray wolves temporally.
Data exclusions	trict some analyses to samples that were higher quality due to the nature of the analysis. For example, for analyses that relied in accurate genotype calls, we restricted these analyses to genomes that were over 20x to ensure reliable calls for heterozygotes, such as for our genetic load analysis .This description of which samples are used from which analysis and why is given in Table S1 and in the methods section.

Reproducibility

We have deposited our newly sequenced genomes to NCBI SRA, in which anyone can download the same raw data to conduct the same analyses in the paper or use it for other analyses. Our scripts are publicly available on this github (https://github.com/hennelly/Asia_wide_wolf_genomics) which has the specific input and flags used for each analysis included in the scripts. The specific version of each statistical program used is given in the methods.

Randomization

For some analyses, such as the genetic load, we analyzed samples according to their geographic location and their clustering within the PCA. For example, we considered wolves from Iran to be one population (Southwest Asia) in our genetic load analysis due to their geographic locations are near each other and each sample clusters closely in the PCA and our Admixture plots.

Blinding

We did not use blinding in our study. Blinding refers to when participants and researchers are unaware of what treatment or intervention the participate is receiving. This type of study design is not applicable to our study, as we do not use humans, live animals, or test subjects.

Did the study involve field work? ☒ Yes ☐ No

Field work, collection and transport

Field conditions

Describe the study conditions for field work, providing relevant parameters (e.g. temperature, rainfall).

Location

State the location of the sampling or experiment, providing relevant parameters (e.g. latitude and longitude, elevation, water depth).

Access & import/export

For collecting wolf samples in Pakistan, we obtained permits from the regional governmental wildlife departments and the CITES permit to collect and export the wolf tissue and blood samples to the USA for sequencing. Wolves are listed as an CITES Appendix 1 species, so we obtained the regional governmental permits as stated previously, the USFWS import permit, and the Ministry of Climate Change of the Government of Pakistan CITES export permit (number P-50/2020). We sequenced these samples in the USA due to no Next Generation sequencing machine being present in Pakistan, however, the lead author generated genetic data and mentored several masters and PhD students in Pakistan on 5 publications where the first author was a Pakistani student. We resequenced the Indian wolf sample BH123 to a higher depth, which this sample was sent for sequencing within India to the company Nucleome. This BH123 sample was caught under a permit by the Government of Maharashtra to Bilal Habib of the Wildlife Institute of India. The wolf sample from Turkey was part of a regional research project on the ecology of wolves under research permits associated (E-21264211-288.04-1602322), and with Turkey's Department of Nature Conservation and National Parks and the Ministry of Agriculture and Forestry's permit for this research (no. 72784983- 488.04-114100). Newly sequenced wolf samples from Slovakia was organized by the State Nature Conservancy of Slovakia, and hunters provided tissue samples to scientists in Slovakia. All other wolf tissue and blood samples were stored at museums and were sampled, collected, and sequenced with permission from those museums.

Disturbance

Describe any disturbance caused by the study and how it was minimized.

Reporting for specific materials, systems and methods

We require information from authors about some types of materials, experimental systems and methods used in many studies. Here, indicate whether each material, system or method listed is relevant to your study. If you are not sure if a list item applies to your research, read the appropriate section before selecting a response.

Materials & experimental systems

n/a	Involved in the study	
☐	☐	Antibodies
☐	☐	Eukaryotic cell lines
☐	☐	Palaeontology and archaeology
☐	☒	Animals and other organisms
☐	☐	Clinical data
☐	☐	Dual use research of concern
☐	☐	Plants

Methods

n/a	Involved in the study	
☐	☐	ChIP-seq
☐	☐	Flow cytometry
☐	☐	MRI-based neuroimaging

Antibodies

Antibodies used

Describe all antibodies used in the study; as applicable, provide supplier name, catalog number, clone name, and lot number.

Validation

Describe the validation of each primary antibody for the species and application, noting any validation statements on the manufacturer's website, relevant citations, antibody profiles in online databases, or data provided in the manuscript.

Eukaryotic cell lines

Policy information about cell lines and Sex and Gender in Research

Cell line source(s)	*State the source of each cell line used and the sex of all primary cell lines and cells derived from human participants or vertebrate models.*
Authentication	*Describe the authentication procedures for each cell line used OR declare that none of the cell lines used were authenticated.*
Mycoplasma contamination	*Confirm that all cell lines tested negative for mycoplasma contamination OR describe the results of the testing for mycoplasma contamination OR declare that the cell lines were not tested for mycoplasma contamination.*
Commonly misidentified lines (See ICLAC register)	*Name any commonly misidentified cell lines used in the study and provide a rationale for their use.*

Palaeontology and Archaeology

Specimen provenance	*Provide provenance information for specimens and describe permits that were obtained for the work (including the name of the issuing authority, the date of issue, and any identifying information). Permits should encompass collection and, where applicable, export.*
Specimen deposition	*Indicate where the specimens have been deposited to permit free access by other researchers.*
Dating methods	*If new dates are provided, describe how they were obtained (e.g. collection, storage, sample pretreatment and measurement), where they were obtained (i.e. lab name), the calibration program and the protocol for quality assurance OR state that no new dates are provided.*

☐ Tick this box to confirm that the raw and calibrated dates are available in the paper or in Supplementary Information.

Ethics oversight	*Identify the organization(s) that approved or provided guidance on the study protocol, OR state that no ethical approval or guidance was required and explain why not.*

Note that full information on the approval of the study protocol must also be provided in the manuscript.

Animals and other research organisms

Policy information about studies involving animals; ARRIVE guidelines recommended for reporting animal research, and Sex and Gender in Research

Laboratory animals	*For laboratory animals, report species, strain and age OR state that the study did not involve laboratory animals.*
Wild animals	For the wild wolf samples in Pakistan, we obtained tissue samples from wolves that were found dead in the field by the government wildlife department staff. One wolf was a captive individual from DG Khan Zoo in Punjab, in which we obtained a blood sample with permission from the Punjab Wildlife Department, which was under the CITES permit for exporting into the US for whole genome sequencing. The wolf from Turkey was captured for a study with required veterinary staff present under research permits associated (E-21264211-288.04-1602322), and with Turkey's Department of Nature Conservation and National Parks and the Ministry of Agriculture and Forestry's permit for this research (no. 72784983- 488.04-114100).
Reporting on sex	*Indicate if findings apply to only one sex; describe whether sex was considered in study design, methods used for assigning sex. Provide data disaggregated for sex where this information has been collected in the source data as appropriate; provide overall numbers in this Reporting Summary. Please state if this information has not been collected. Report sex-based analyses where performed, justify reasons for lack of sex-based analysis.*
Field-collected samples	For samples collected in the field from dead carcasses, a small piece of tissue was cut from the carcass (usually the ear) and stored in a small tube filled with ethanol or kept cool and dry if no ethanol was present. The tube was placed in a freezer until DNA extraction could take place. For wild wolves that were captured in the field (for example the wolf from Turkey) a blood sample was taken and stored in a purple top blood EDTA vial, kept frozen until sequencing.
Ethics oversight	No ethical approval was needed as our study did not involve experiments on live animals.

Note that full information on the approval of the study protocol must also be provided in the manuscript.

Clinical data

Policy information about clinical studies

All manuscripts should comply with the ICMJE guidelines for publication of clinical research and a completed CONSORT checklist must be included with all submissions.

Clinical trial registration	*Provide the trial registration number from ClinicalTrials.gov or an equivalent agency.*

Study protocol | *Note where the full trial protocol can be accessed OR if not available, explain why.*

Data collection | *Describe the settings and locales of data collection, noting the time periods of recruitment and data collection.*

Outcomes | *Describe how you pre-defined primary and secondary outcome measures and how you assessed these measures.*

Dual use research of concern

Policy information about dual use research of concern

Hazards

Could the accidental, deliberate or reckless misuse of agents or technologies generated in the work, or the application of information presented in the manuscript, pose a threat to:

No	Yes	
☒	☐	Public health
☒	☐	National security
☒	☐	Crops and/or livestock
☒	☐	Ecosystems
☒	☐	Any other significant area

Experiments of concern

Does the work involve any of these experiments of concern:

No	Yes	
☒	☐	Demonstrate how to render a vaccine ineffective
☒	☐	Confer resistance to therapeutically useful antibiotics or antiviral agents
☒	☐	Enhance the virulence of a pathogen or render a nonpathogen virulent
☒	☐	Increase transmissibility of a pathogen
☒	☐	Alter the host range of a pathogen
☒	☐	Enable evasion of diagnostic/detection modalities
☒	☐	Enable the weaponization of a biological agent or toxin
☒	☐	Any other potentially harmful combination of experiments and agents

Plants

Seed stocks | *Report on the source of all seed stocks or other plant material used. If applicable, state the seed stock centre and catalogue number. If plant specimens were collected from the field, describe the collection location, date and sampling procedures.*

Novel plant genotypes | *Describe the methods by which all novel plant genotypes were produced. This includes those generated by transgenic approaches, gene editing, chemical/radiation-based mutagenesis and hybridization. For transgenic lines, describe the transformation method, the number of independent lines analyzed and the generation upon which experiments were performed. For gene-edited lines, describe the editor used, the endogenous sequence targeted for editing, the targeting guide RNA sequence (if applicable) and how the editor was applied.*

Authentication | *Describe any authentication procedures for each seed stock used or novel genotype generated. Describe any experiments used to assess the effect of a mutation and, where applicable, how potential secondary effects (e.g. second site T-DNA insertions, mosiacism, off-target gene editing) were examined.*

ChIP-seq

Data deposition

☐ Confirm that both raw and final processed data have been deposited in a public database such as GEO.

☐ Confirm that you have deposited or provided access to graph files (e.g. BED files) for the called peaks.

Data access links
May remain private before publication. | *For "Initial submission" or "Revised version" documents, provide reviewer access links. For your "Final submission" document, provide a link to the deposited data.*

Files in database submission | *Provide a list of all files available in the database submission.*

Genome browser session
(e.g. UCSC)

Provide a link to an anonymized genome browser session for "Initial submission" and "Revised version" documents only, to enable peer review. Write "no longer applicable" for "Final submission" documents.

Methodology

Replicates

Describe the experimental replicates, specifying number, type and replicate agreement.

Sequencing depth

Describe the sequencing depth for each experiment, providing the total number of reads, uniquely mapped reads, length of reads and whether they were paired- or single-end.

Antibodies

Describe the antibodies used for the ChIP-seq experiments; as applicable, provide supplier name, catalog number, clone name, and lot number.

Peak calling parameters

Specify the command line program and parameters used for read mapping and peak calling, including the ChIP, control and index files used.

Data quality

Describe the methods used to ensure data quality in full detail, including how many peaks are at FDR 5% and above 5-fold enrichment.

Software

Describe the software used to collect and analyze the ChIP-seq data. For custom code that has been deposited into a community repository, provide accession details.

Flow Cytometry

Plots

Confirm that:

☐ The axis labels state the marker and fluorochrome used (e.g. CD4-FITC).

☐ The axis scales are clearly visible. Include numbers along axes only for bottom left plot of group (a 'group' is an analysis of identical markers).

☐ All plots are contour plots with outliers or pseudocolor plots.

☐ A numerical value for number of cells or percentage (with statistics) is provided.

Methodology

Sample preparation

Describe the sample preparation, detailing the biological source of the cells and any tissue processing steps used.

Instrument

Identify the instrument used for data collection, specifying make and model number.

Software

Describe the software used to collect and analyze the flow cytometry data. For custom code that has been deposited into a community repository, provide accession details.

Cell population abundance

Describe the abundance of the relevant cell populations within post-sort fractions, providing details on the purity of the samples and how it was determined.

Gating strategy

Describe the gating strategy used for all relevant experiments, specifying the preliminary FSC/SSC gates of the starting cell population, indicating where boundaries between "positive" and "negative" staining cell populations are defined.

☐ Tick this box to confirm that a figure exemplifying the gating strategy is provided in the Supplementary Information.

Magnetic resonance imaging

Experimental design

Design type

Indicate task or resting state; event-related or block design.

Design specifications

Specify the number of blocks, trials or experimental units per session and/or subject, and specify the length of each trial or block (if trials are blocked) and interval between trials.

Behavioral performance measures

State number and/or type of variables recorded (e.g. correct button press, response time) and what statistics were used to establish that the subjects were performing the task as expected (e.g. mean, range, and/or standard deviation across subjects).

Acquisition

Imaging type(s) — *Specify: functional, structural, diffusion, perfusion.*

Field strength — *Specify in Tesla*

Sequence & imaging parameters — *Specify the pulse sequence type (gradient echo, spin echo, etc.), imaging type (EPI, spiral, etc.), field of view, matrix size, slice thickness, orientation and TE/TR/flip angle.*

Area of acquisition — *State whether a whole brain scan was used OR define the area of acquisition, describing how the region was determined.*

Diffusion MRI ☐ Used ☐ Not used

Preprocessing

Preprocessing software — *Provide detail on software version and revision number and on specific parameters (model/functions, brain extraction, segmentation, smoothing kernel size, etc.).*

Normalization — *If data were normalized/standardized, describe the approach(es): specify linear or non-linear and define image types used for transformation OR indicate that data were not normalized and explain rationale for lack of normalization.*

Normalization template — *Describe the template used for normalization/transformation, specifying subject space or group standardized space (e.g. original Talairach, MNI305, ICBM152) OR indicate that the data were not normalized.*

Noise and artifact removal — *Describe your procedure(s) for artifact and structured noise removal, specifying motion parameters, tissue signals and physiological signals (heart rate, respiration).*

Volume censoring — *Define your software and/or method and criteria for volume censoring, and state the extent of such censoring.*

Statistical modeling & inference

Model type and settings — *Specify type (mass univariate, multivariate, RSA, predictive, etc.) and describe essential details of the model at the first and second levels (e.g. fixed, random or mixed effects; drift or auto-correlation).*

Effect(s) tested — *Define precise effect in terms of the task or stimulus conditions instead of psychological concepts and indicate whether ANOVA or factorial designs were used.*

Specify type of analysis: ☐ Whole brain ☐ ROI-based ☐ Both

Statistic type for inference — *Specify voxel-wise or cluster-wise and report all relevant parameters for cluster-wise methods.*

(See Eklund et al. 2016)

Correction — *Describe the type of correction and how it is obtained for multiple comparisons (e.g. FWE, FDR, permutation or Monte Carlo).*

Models & analysis

n/a	Involved in the study
☐	☐ Functional and/or effective connectivity
☐	☐ Graph analysis
☐	☐ Multivariate modeling or predictive analysis

Functional and/or effective connectivity — *Report the measures of dependence used and the model details (e.g. Pearson correlation, partial correlation, mutual information).*

Graph analysis — *Report the dependent variable and connectivity measure, specifying weighted graph or binarized graph, subject- or group-level, and the global and/or node summaries used (e.g. clustering coefficient, efficiency, etc.).*

Multivariate modeling and predictive analysis — *Specify independent variables, features extraction and dimension reduction, model, training and evaluation metrics.*
